# Klf9 Loss of Function Protects Against Glucocorticoids Induced Skeletal Muscle Wasting

**DOI:** 10.1002/jcsm.70020

**Published:** 2025-07-28

**Authors:** Yujie Zhang, Jingran Hao, Yueyao Feng, Tongtong Qiu, Jinjin Wu, Xuenan Zhou, Heng Fan, Yongsheng Chang

**Affiliations:** ^1^ Key Laboratory of Immune Microenvironment and Disease (Ministry of Education), Tianjin Key Laboratory of Cellular Homeostasis and Disease, Department of Physiology and Pathophysiology Tianjin Medical University Tianjin China; ^2^ Department of Reproductive Medicine General Hospital of Ningxia Medical University (The First Clinical Medical College of Ningxia Medical University) Yinchuan China; ^3^ Ningxia Key Laboratory of Stem Cell and Regenerative Medicine, Institute of Medical Sciences, General Hospital of Ningxia Medical University Yinchuan China

**Keywords:** glucocorticoids, Krüppel‐like factor 9, muscle atrophy, myostatin

## Abstract

**Background:**

Glucocorticoids (GCs) are the most important and frequently used class of anti‐inflammatory drugs. However, the mechanisms underlying excessive glucocorticoid‐mediated induction of muscle atrophy remain incompletely understood.

**Methods:**

We generated skeletal muscle‐specific *Klf9* transgenic mice (m*Klf9*TG) and skeletal muscle‐specific *Klf9* knockout mice (*Klf9* ^mlc−/−^). The body weight, tissue weight, body composition, grip strength, running distance and muscle fibre cross section of m*Klf9*TG, *Klf9* ^mlc−/−^ mice and their littermate controls were examined. Expression of genes related to muscle protein synthesis and degradation pathways were also tested in the m*Klf9*TG mice, *Klf9* ^mlc−/−^ mice and their littermate controls. We performed Klf9 gain‐ or loss‐of‐function studies in differentiated C2C12 myotubes using lentiviruses encoding *Klf9* or the shRNA specific to *Klf9* in vitro. Luciferase reporter gene assay and ChIP assay were performed to explore the molecular mechanism of Klf9 action. *Klf9* ^mlc−/−^ and *Klf9* ^fl/fl^ mice were treated with dexamethasone (Dex). Multiple genetic and pharmacological approaches were also used to investigate the intracellular signalling cascades underlying the Dex/Klf9‐ediated skeletal muscle wasting.

**Results:**

Skeletal muscle *Klf9* gene expression was significantly upregulated by Dex (*p* < 0.05 or *p* < 0.01 vs. vehicle group). Compared with littermate control mice (R‐loxP), m*Klf9*TG mice exhibited decreased skeletal muscle mass (TA 0.101 ± 0.018 vs. 0.040 ± 0.007 g, *p* < 0.001) and impaired grip strength (forelimb 157.4 ± 3.7 vs. 93.45 ± 9.8 and four limbs 255.3 ± 23.1 vs. 170.1 ± 36.2, *p* < 0.001). Conversely, compared with *Klf9* ^fl/fl^, *Klf9* ^mlc−/−^ mice exhibited increased skeletal muscle mass (TA 0.103 ± 0.012 vs. 0.123 ± 0.005 g, *p* < 0.001) and enhanced grip strength (forelimb 110.3 ± 5.8 vs. 156.8 ± 10.0 and four limbs 155.5 ± 6.3 vs. 226.5 ± 19.7, *p* < 0.001). Skeletal muscle Klf9 deficiency alleviated muscle atrophy induced by acute high‐dose Dex treatment (*p* < 0.001). Mechanistically, Klf9 induces the expression of myostatin (Mstn) and muscle atrophy F‐box (MAFbx) by directly binding to and activating the transcription of their promoters. Treatment of AAV‐MSTN reduced the increased grip strength of *Klf9* ^mlc−/−^ mice (forelimb 143.5 ± 22.3 vs. 118.8 ± 3.1 and four limbs 249.8 ± 24.7 vs. 208.7 ± 9.0, *p* < 0.001).

**Conclusions:**

In summary, our study provides novel insights into the mechanisms underlying GC‐induced muscular atrophy and reveals that skeletal muscle induction of Klf9 expression is a mechanism underlying GC therapy‐induced muscle loss. Thus, targeting Klf9 may offer novel approaches to the treatment of skeletal muscle wasting diseases.

## 1 Background

Skeletal muscle is the largest organ, which constitutes 50%–70% of the protein content of the body and has multiple functions, including movement, thermostasis, glucose and lipid metabolism. The control of muscle mass is essential for muscle strength and physical activity [1].

Synthetic glucocorticoids (GCs), including Dex, are commonly used to treat various inflammatory illnesses. However, their chronic use causes several side effects, including insulin resistance, hypertension, reduced body weight and muscle atrophy in humans and animals [2]. The GC receptor (GR) is mandatory for muscle atrophy in response to excess GC. Muscle‐specific GR‐knockout mice are resistant to the atrophy‐inducing effects of GCs [3]. Current evidence suggests that GCs induce muscle atrophy by increasing the rate of protein degradation via the ubiquitin‐proteasome system and the autophagy‐lysosome system. Moreover, protein synthesis is also inhibited by glucocorticoid treatment [4]. Glucocorticoid stimulation of the ubiquitin–proteasome system is mediated through the increased expression of atrophy‐related genes, including MAFbx and tripartite motif‐containing 63 (MuRF1), two muscle‐specific E3 ligases implicated in the regulation of skeletal muscle atrophy under various physiological and pathological conditions. GCs stimulate the conversion of microtubule‐associated protein 1 light chain 3 (LC3)‐I to LC3‐II, an indicator of autophagy [5].

GCs have been demonstrated to inhibit the muscle hypertrophic pathway, including the PI3K/AKT signalling pathway [6]. The inhibition of protein synthesis by GCs results mainly from the inhibition of mTORC1, the kinase responsible for the phosphorylation of the translation inhibitor eukaryotic initiation factor‐4E (4EBP1) and p70 ribosomal protein S6 kinase (P70S6K1) [7].

GCs can also induce muscle atrophy by stimulating the production of MSTN in muscle [8, 9]. Myostatin (Mstn) has been shown to be the master negative regulator of skeletal muscle mass [10]. Upon activation by further proteolytic cleavage of the propeptide, the mature MSTN dimer binds directly to activin receptor IIA (ACVR2A) and activin receptor IIB (ACVR2B), after which activin A receptor type 1B (ALK4) and transforming growth factor beta receptor I (ALK5) are engaged, followed by the activation of SMAD2 and SMAD3 to induce muscle atrophy. Mstn decreases protein synthesis by inhibiting the AKT/mTOR signalling pathway and inducing muscle atrophy by activating the transcription of atrophy‐related genes, including MAFbx and MuRF1 [11, 12, 13].

Klf9, a member of the Krüppel‐like family of zinc‐finger domain transcription factors, plays a key role in development [14]. Our previous studies demonstrated that Klf9 regulates hepatic gluconeogenesis and thermogenesis in brown and beige fat [15, 16]. Klf9 is ubiquitously expressed in many tissues [17].

In the present study, we showed that Klf9 is induced by Dex in skeletal muscle. The skeletal muscle‐specific *Klf9* transgene induced muscle atrophy. Conversely, *Klf9* knockout promoted muscle hypertrophy. Our study reveals that Klf9 is a novel regulator of skeletal muscle mass and provides novel insight into the molecular mechanism of GC‐induced muscle atrophy.

## 2 Methods

### 2.1 Animal Studies

*Klf9* ^fl/fl^ and *Klf9 Rosa26* knock‐in (*Rosa26‐*LSL*‐Klf9* ^flox/flox^, R‐loxP) mice were generated by the CRISPR/Cas9 system at Beijing Biocytogen Co. Ltd. as previously described [15]. To generate skeletal muscle‐specific Klf9 knockout mice, *Klf9* ^fl/fl^ mice were crossed with MLC‐Cre, *Klf9* ^fl/fl^ mice to obtain MLC‐Cre, *Klf9* ^fl/fl^ mice (called *Klf9* ^mlc−/−^). The *Klf9* ^fl/fl^ littermates were used as controls. R‐loxP mice were crossed with MLC‐Cre to obtain skeletal muscle‐specific overexpression Klf9 mice: MLC‐Cre, R‐loxP mice (called m*Klf9*TG). Littermates lacking the MLC‐Cre gene (R‐loxP mice) were used as controls.

### 2.2 Exercise Stress Test

Eight‐week old male C57BL/6J WT mice were individually housed in cages with free access to a running wheel (free wheel running). Mice were exercised for 30 days and sacrificed 4 h after the last running.

### 2.3 Histology Analysis

For H&E staining, immunohistochemistry, Oil Red O staining and NADH‐TR staining, tissues were treated as described previously [18, 19].

### 2.4 Autophagic Flux Analysis

Autophagic flux was monitored in vivo muscle using colchicine (MCE, HY‐16569) as previously described [20]. m*Klf9*TG mice, *Klf9* ^mlc−/−^ mice and their littermate controls were i.p. injected with vehicle or with 0.4 mg/kg colchicine. Colchicine was administered twice, at 24 and 12 h before muscle dissection.

### 2.5 Metabolite Measurement

Serum triacylglycerol, total cholesterol levels, hepatic concentrations of triacylglycerol and total cholesterol were quantified with enzymatic assays (Applygen, China) according to the manufacturer's instructions. Serum CK concentration was determined using an automated Monarch device (Large research equipment sharing platform of Tianjin Medical University).

### 2.6 ChIP Assay

Briefly, C2C12 cells were infected with Lenti‐GFP or Lenti‐*Klf9* for 72 h and then were harvested for ChIP assay as described previously [16]. Antibodies specific for Myc (Immunoway, YN5506) or unspecific IgG (sc‐2027; Santa Cruz) were used for ChIP assay. The purified DNA was used to amplify the KLF9 regulatory element on the mouse Mstn promoter by a real‐time PCR reaction. Primers directed at upstream or downstream of the binding site were used as a negative control. The sequences of primers are shown in Table S1.

### 2.7 Additional Methods

Additional details regarding the methods and materials are provided in the Supporting Information.

### 2.8 Statistics

The quantitative data are represented as the mean ± the standard error of the mean (SEM) of three independent experiments. In most of the cases for in vivo experiments in mice, an *n* = 6 was the minimum amount used. A two‐tailed, unpaired Student's *t* test was used for pairwise comparison of genotypes or treatments. One‐way ANOVA and two‐way ANOVA were used when comparing three or more groups, as indicated in the figure legends and otherwise. Analysis was performed using Microsoft Excel and/or GraphPad Prism. *p* < 0.05 was considered significant, as indicated by asterisks in the figure legends.

## 3 Results

### 3.1 Skeletal Muscle Klf9 Expression Is Regulated by Dex and Exercise

**FIGURE 1:**
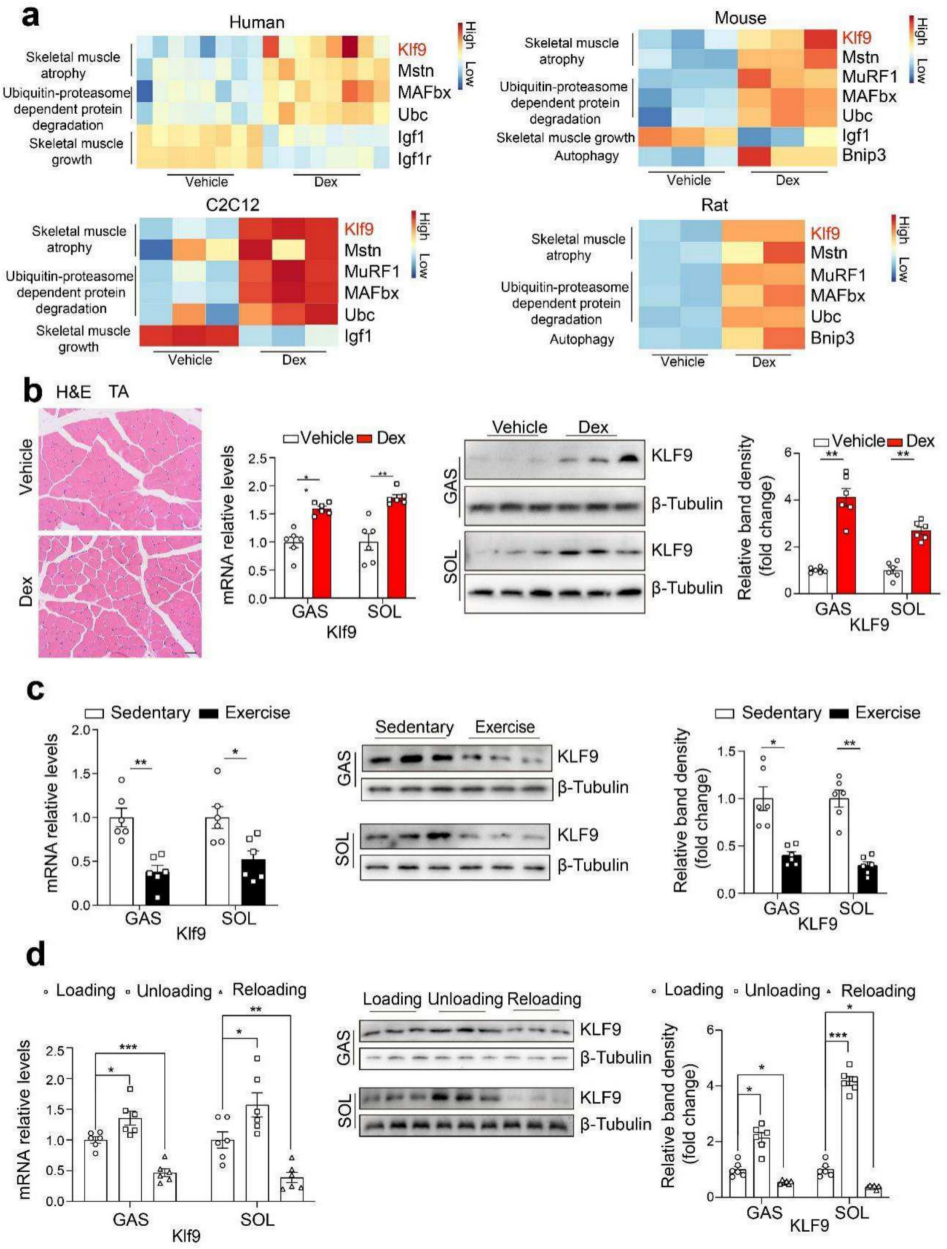
Skeletal muscle Klf9 expression is regulated by Dex and exercise. (a) Heat map representation of the atrophy related genes expression in skeletal muscle biopsies from male healthy volunteers with dexamethasone (Dex) at 4 mg/kg/day or vehicle treatments for 4 days (GSE17992) (upper left) (*n* = 8/group). Heat map representation of the atrophy related genes expression in skeletal muscle from mice intraperitoneally injected with 2 mg/kg Dex or vehicle for 8 h (GSE74624) (upper right) (*n* = 3/group). Heat map representation of the atrophy related genes expression in C2C12 myotubes under 200‐μM Dex or vehicle for 24 h (GSE261787) (lower left) (*n* = 3/group). Heat map representation of the atrophy related genes and Klf9 expression in skeletal muscle from rats (250‐g males) administered with dexamethasone (0.7 mg/kg/day) or vehicle by continuous infusion (Alzet pumps) for 7 days (GSE12296) (lower right) (*n* = 2/group). (b) Haematoxylin and eosin staining of paraffin‐embedded TA sections from mice intraperitoneally injected with Dex at 25 mg/kg/day for 9 days (left). Quantitative PCR analysis of Klf9 in skeletal muscle from the indicated mice (middle). Representative Western blotting analysis of protein levels of Klf9 in skeletal muscle of the indicated mice. Protein amount was quantified using Image J (right) (*n* = 6/group). (c) qPCR analysis of Klf9 expression in skeletal muscle of mice in sedentary and exercise groups (left). Male C57BL/6J wild‐type (WT) mice were individually housed in cages with access to a running wheel (free wheel‐running) for 30 days and sacrificed after 1‐h treadmill exercise. Representative Western blotting analysis of protein levels of Klf9 in skeletal muscle of WT mice in sedentary and exercise groups. Protein amount was quantified using Image J (right) (*n* = 6/group). (d) qPCR analysis of Klf9 expression in skeletal muscle of WT mice in loading control (nonsuspension), unloading (suspension for 10 days), and reloading (4 days reload after 10 days suspension) (left) Representative Western blotting analysis of protein levels of Klf9 in skeletal muscle of WT mice in loading, unloading and reloading groups. Protein amount was quantified using Image J (right) (*n* = 6/group). All data are shown as mean ± SEM. Unpaired two‐tailed Student's *t* tests were performed in (b) and (c). Two‐way analysis of variance (ANOVA) was performed in d. Scale bars: 20 μm.

We first analysed the publicly available transcriptome datasets GSE17992 (human skeletal muscle biopsies from healthy male volunteers treated with Dex for 4 days) [21], GSE74624 (skeletal muscle from mice injected with 2‐mg/kg Dex) [22], GSE261787 (C2C12 myotubes treated with 200‐μM Dex for 24 h) and GSE12296 (gastrocnemius (GAS) skeletal muscle from rats administered Dex) [23]. Notably, we found that Klf9 expression was upregulated in the skeletal muscle of humans, mice and rats, as well as C2C12 cells, by Dex treatment (Figure 1a). Thus, we tested Klf9 expression in high‐dose Dex‐treated mouse skeletal muscle. H&E staining revealed that the myofiber cross‐sectional area was decreased in Dex‐treated skeletal muscle (Figure 1b). The real‐time PCR and Western blotting results indicated that Dex induces Klf9 expression in the GAS and soleus (SOL) muscles (Figure 1b). Furthermore, Klf9 expression was downregulated in the skeletal muscle of trained mice (Figure 1c). In addition, unloading induced an increase in the mRNA and protein levels of Klf9 in skeletal muscle, whereas reloading reversed this increase (Figure 1d).

### 3.2 Skeletal Muscle‐Specific Transgenic *Klf9* Expression Decreases Skeletal Muscle Mass

**FIGURE 2:**
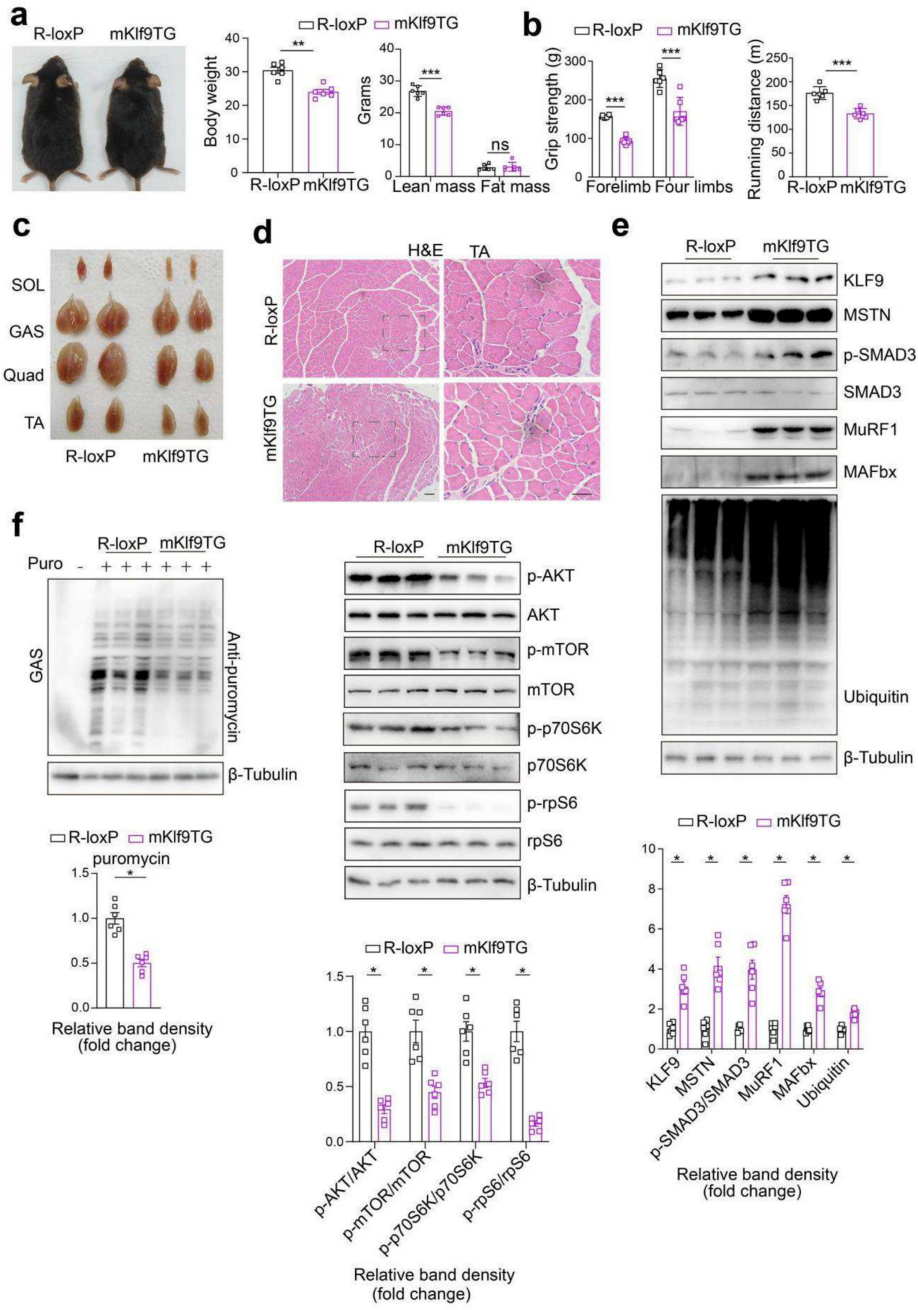
Skeletal muscle‐specific transgenic *Klf9* expression decreases skeletal muscle mass. (a) Gross morphology of chow diet‐fed R‐loxP and m*Klf9*TG mice at 3 months of age (left). Body weight of R‐loxP and m*Klf9*TG mice (middle) (*n* = 6/group). MRI analysis of body composition of the indicated mice (right) (*n* = 6/group). (b) Forelimb and four limbs grip strength was tested in 3 month‐old R‐loxP and m*Klf9*TG mice (left) (*n* = 6/group). Mean running distance on a motorized treadmill for mice of the indicated genotypes at 3 months of age (right) (*n* = 6/group). (c) Gross appearance of soleus (SOL), gastrocnemius (GAS), quadriceps (QUA) and tibialis anterior (TA) from R‐loxP and m*Klf9*TG mice described in (a). (d) Haematoxylin and eosin staining of paraffin‐embedded TA sections of mice described in (a). (e) Representative Western blotting analysis of KLF9, MSTN, p‐SMAD3, total‐SMAD3, MuRF1, MAFbx and Ubiquitin levels in skeletal muscle from mice in (a) (*n* = 6/group). (f) Representative Western blotting analysis of puromycin incorporation in gastrocnemius from indicated mice at the age of 3 months (left). in vivo protein synthesis rates were measured via the SUnSET technique. Representative Western blotting analysis for protein synthesis related protein levels in gastrocnemius from mice in (a) (right). Quantification of the KLF9/Tubulin, MSTN/Tubulin, MuRF1/Tubulin, MAFbx/Tubulin, Ubiquitin/Tubulin, p‐SMAD3/SMAD3, Puromycin/Tubulin p‐RPS6/RPS6, p‐p70S6K/p70S6K, p‐AKT/AKT and p‐mTOR/mTOR signal ratios (bottom) (*n* = 6/group). All data are shown as mean ± SEM. Unpaired two‐tailed Student's *t* tests were performed in (a), (b), (e) and (f). Scale bars: 20 μm.

To explore the physiological function of Klf9 in skeletal muscle, we generated skeletal muscle‐specific *Klf9* transgenic (m*Klf9*TG) mice (Figure S1A–C) [24, 25]. Under a normal chow diet, compared with control mice, *Klf9* transgenic mice gained less body weight as they aged, although they consumed an amount of food similar to that of the controls (Figures 2a and S1D). MRI confirmed that m*Klf9*TG mice had reduced lean mass, whereas fat mass did not differ between m*Klf9*TG mice and controls (Figure 2a). The grip strength of adult m*Klf9*TG mice was significantly reduced (Figure 2b). Acute running endurance tests revealed that the maximal running capacity of m*Klf9*TG mice was diminished (Figure 2b). Thus, Klf9 overexpression in skeletal muscle caused a sharp decrease in muscle mass and muscle weakness, as well as impaired exercise performance. The size and weight of skeletal muscles, which differ in their predominant fibre types, including the SOL, quadriceps (Quad), tibialis anterior (TA) and GAS, were decreased in m*Klf9*TG mice at 3 months of age (Figures 2c and S1E). Moreover, the skeletal muscle‐specific *Klf9* transgene did not affect the size or weight of brown adipose tissue (BAT), inguinal WAT (iWAT), epididymal WAT (eWAT) or liver under chow diet conditions (Figure S1F).

Consistently, H&E staining revealed a remarkable decrease in myofiber size in m*Klf9*TG mice compared with that observed in R‐loxP controls (Figures 2d and S1G). These data clearly demonstrate that the *Klf9* transgene led to myofiber atrophy.

To elucidate the underlying molecular mechanisms of Klf9‐mediated muscle atrophy, we performed transcriptomic analysis of muscle from 3‐month‐old control and m*Klf9*TG mice. Preliminary analysis of the RNA‐seq data revealed 272 downregulated and 2424 upregulated genes (Figure S1H). Kyoto Encyclopedia of Genes and Genomes (KEGG) data analysis revealed that the upregulated genes were significantly enriched in functionally annotated pathways, including the ubiquitin‐mediated proteolysis and autophagy‐lysosome pathways. In contrast, the downregulated genes were enriched in fatty acid metabolism and protein synthesis pathways (Figure S1I). Moreover, a heatmap revealed that the overexpression of Klf9 resulted in the marked upregulation of the expression of genes involved in ubiquitin‐mediated proteolysis and autophagy, whereas it decreased the expression of genes involved in mitochondrial energy metabolism, fatty acid oxidation, and muscle structural proteins (Figure S1J). Notably, we observed that Klf9 regulated the expression of MuRF1 and MAFbx. In addition, the expression of myostatin was also induced (Figure S1K). Furthermore, real‐time PCR and Western blotting confirmed that the overexpression of Klf9 in mice activated the skeletal muscle atrophy programme. The mRNA and protein levels of atrophy‐related genes, including Mstn, MuRF1 and MAFbx, were increased in the GAS muscle of m*Klf9*TG mice (Figures 2e and S1L). Correspondingly, the phosphorylation of SMAD3, the downstream transcription factor of myostatin, was increased, and the content of ubiquitinated proteins in muscle homogenates from m*Klf9*TG mice was increased (Figure 2e). Both the ubiquitin–proteasome system and the autophagy‐lysosome system are implicated in the skeletal muscle atrophy induced by GCs and myostatin [4, 26]. To confirm the activated autophagy programme in m*Klf9*TG muscle, we assessed autophagic flux by treating mice with colchicine, an established inhibitor of lysosomal degradation. As a result, the Klf9 transgene led to a greater accumulation of LC3‐II protein in whole muscle homogenates in the presence or absence of colchicine treatment (Figure S1M), indicating an enhanced general autophagic flux in m*Klf9*TG muscles.

The control of skeletal muscle mass is determined by the dynamic balance between anabolic and catabolic processes [27, 28]. Myostatin has been shown to inhibit skeletal muscle protein synthesis. We monitored the protein synthesis rate in vivo by using the SUnSET assay, which involves labelling nascent proteins with puromycin to monitor protein translation efficiency in muscle. This assay revealed reduced levels of puromycin‐labelled nascent polypeptide chains in the muscle of m*Klf9*TG mice (Figure 2f). Moreover, the phosphorylation levels of signalling molecules controlling muscle protein synthesis, including AKT, p70S6K, mTOR and rpS6, were decreased in the skeletal muscle of m*Klf9*TG mice (Figure 2f). Thus, a reduction in the rate of protein synthesis also contributed to the skeletal muscle atrophy present in m*Klf9*TG mice.

These data indicate that skeletal muscle Klf9 overexpression enhances ubiquitin‐mediated protein degradation and autophagy and inhibits protein synthesis, thereby leading to skeletal muscle atrophy.

In addition, our real‐time PCR data confirmed that Klf9 overexpression in skeletal muscle decreased the expression of genes involved in fatty acid oxidation and mitochondrial biogenesis (Figure S1N), thereby decreasing the mtDNA copy number (Figure S1O). Transmission electron microscopy revealed that the number of larger mitochondria was decreased in the soleus of m*Klf9*TG mice (Figure S1P).

The fibre type composition was examined in the SOL and TA muscle of the mice. Nicotinamide adenine dinucleotide dehydrogenase‐tetrazolium reductase (NADH‐TR) staining confirmed a marked decrease in the percentage of oxidative fibres in the SOL and TA muscles of m*Klf9*TG mice (Figure S1Q). The expression of genes encoding the major oxidative myosin isoforms, including type I MHC1 (Myh7 gene) and type IIa MHC2A (Myh2 gene), was reduced in mKlf9TG muscles. In contrast, the expression of the genes encoding the major glycolytic type IIx myosin isoform MHCIIx (Myh1 gene) and type IIb myosin isoform MHC2B (Myh4 gene) was increased in mKlf9TG muscles (Figure S1R).

### 3.3 Skeletal Muscle‐Specific *Klf9* Deficiency in Mice Leads to Increased Muscle Mass

**FIGURE 3:**
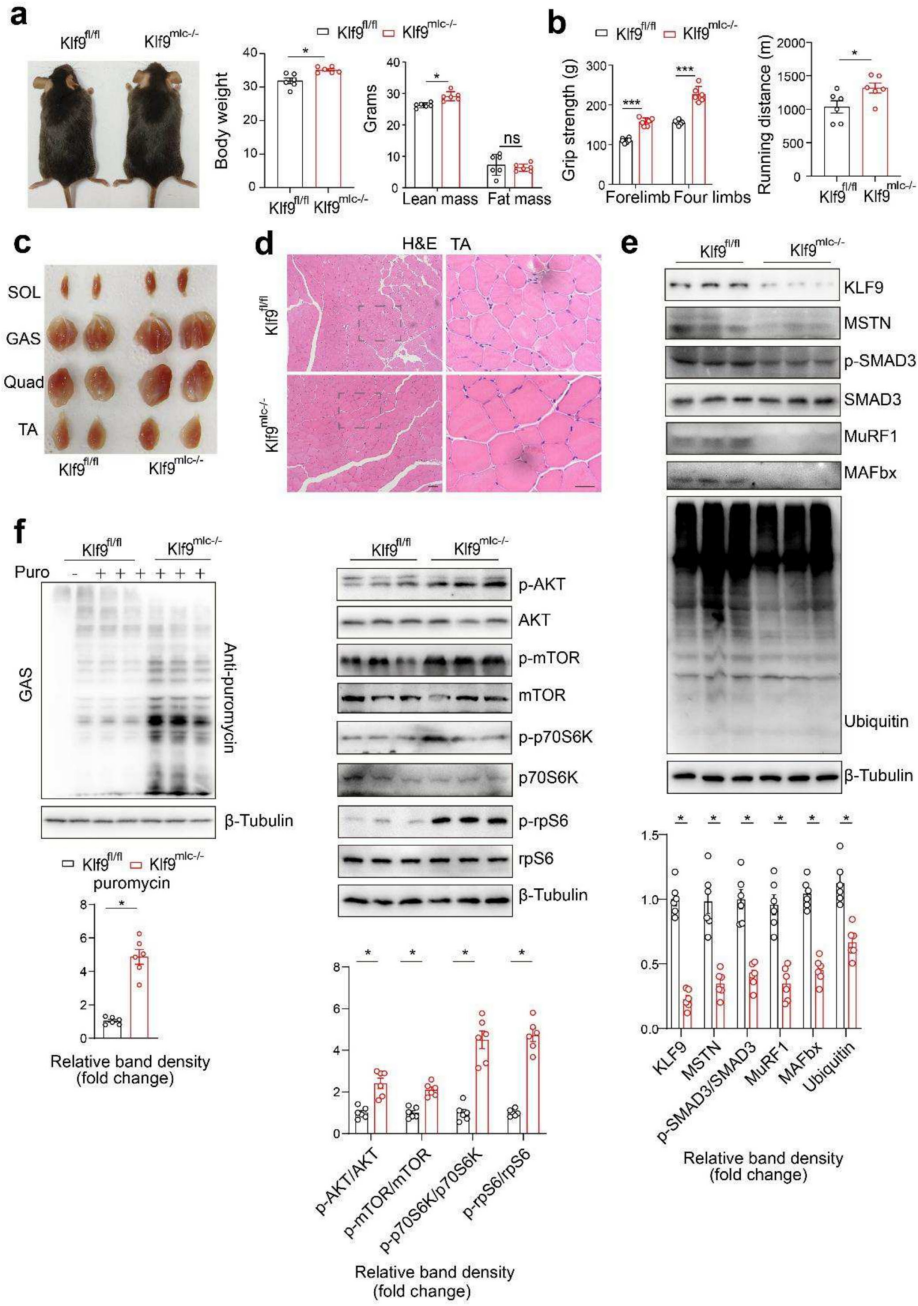
Skeletal muscle‐specific Klf9 deficiency in mice leads to increased muscle mass. (a) Gross morphology of chow diet‐fed *Klf9* ^fl/fl^ and *Klf9* ^mlc−/−^ mice at 4 months of age (left). Body weight of *Klf9* ^fl/fl^ and *Klf9* ^mlc−/−^ mice (middle) (*n* = 6/group). MRI analysis of body composition of the indicated mice (right) (*n* = 6/group). (b) Forelimb and four limbs grip strength was tested in 4 month‐old *Klf9* ^fl/fl^ and *Klf9* ^mlc−/−^ mice (left) (*n* = 6/group). Mean running distance on a motorized treadmill for mice of the indicated genotypes at 4 months of age (right) (*n* = 6/group). (c) Gross appearance of soleus (SOL), gastrocnemius (GAS), quadriceps (QUA) and tibialis anterior (TA) from *Klf9* ^fl/fl^ and *Klf9* ^mlc−/−^ mice described in a. (d) Haematoxylin and eosin staining of paraffin‐embedded TA sections of mice described in (a). (e) Representative Western blotting analysis of KLF9, MSTN, p‐SMAD3, total‐SMAD3, MuRF1, MAFbx and Ubiquitin levels in skeletal muscle from mice in (a). (f) Representative Western blotting analysis of puromycin incorporation in gastrocnemius from indicated mice at the age of 4 months (left). in vivo protein synthesis rates were measured via the SUnSET technique. Representative Western blotting analysis for protein synthesis related protein levels in gastrocnemius from mice in (a) (right). Quantification of the KLF9/Tubulin, MSTN/Tubulin, MuRF1/Tubulin, MAFbx/Tubulin, p‐SMAD3/SMAD3, Ubiquitin/Tubulin, Puromycin/Tubulin, p‐RPS6/RPS6, p‐p70S6K/p70S6K, p‐AKT/AKT and p‐mTOR/mTOR signal ratios (bottom) (*n* = 6/group). All data are shown as mean ± SEM. Unpaired two‐tailed Student's *t* tests were performed in (a), (b), (e) and (f). Scale bars: 20 μm.

We generated a skeletal muscle‐specific *Klf9* knockout mouse model to conduct Klf9 loss‐of‐function studies by crossing MLC‐Cre mice with *Klf9* ^fl/fl^ mice [15, 24]. The resulting MLC‐Cre; *Klf9* ^fl/fl^ mice are henceforth referred to as *Klf9* ^mlc−/−^ mice (Figure S2A‐C). *Klf9* ^mlc−/−^ mice gained more weight than control mice did, although they consumed a similar amount of food as the control mice did (Figure 3a and S2D). MRI confirmed that *Klf9* ^mlc−/−^ mice at 4 months of age had more lean mass than control mice did while fat mass remained unaltered (Figure 3a). Moreover, the grip strength of the adult *Klf9* ^mlc−/−^ mice was significantly greater than that of the control mice. Acute running endurance tests revealed that the maximal running capacity of *Klf9* ^mlc−/−^ mice was greater than that of control mice (Figure 3b). Indeed, the size and weight of skeletal muscle, including the SOL, Quad, TA and GAS, were increased in *Klf9* ^mlc−/−^ mice (Figures 3c and S2E).

However, Klf9 deficiency in skeletal muscle did not affect the weights of the liver, BAT, WAT or other tissues examined (Figure S2F). H&E staining revealed a remarkable increase in the size of myofibers in *Klf9* ^mlc−/−^ mice (Figures 3d and S2G). These data clearly indicate that the loss of function of Klf9 in skeletal muscle promotes myofiber hypertrophy.

Consistently, the mRNA and protein levels of atrophy‐related genes were reduced in the GAS muscle of *Klf9* ^mlc−/−^ mice (Figures 3e and S2H). The content of ubiquitinated proteins in muscle homogenates from *Klf9* ^mlc−/−^ mice was decreased (Figure 3e). Moreover, Klf9 deficiency also inhibited the general autophagic flux in GAS muscle (Figure S2I). The muscle protein synthesis and its related signalling pathways were activated in the GAS muscle of *Klf9* ^mlc−/−^ mice compared with the controls (Figure 3f). Thus, Klf9 deficiency in skeletal muscle inhibited protein degradation and enhanced protein synthesis, thereby increasing muscle mass and physiological function.

Klf9 deficiency in the skeletal muscle of mice also increased the expression of genes involved in fatty acid oxidation and mitochondrial biogenesis (Figure S2J), thereby increasing the mtDNA copy number (Figure S2K). Transmission electron microscopy revealed that *Klf9* knockout increased the number of mitochondria with larger sizes (Figure S2L). Thus, Klf9 deficiency in skeletal muscle improved mitochondrial energy metabolism. The NADH‐TR staining and the assessment of the expression of muscle fibre‐selective myosin isoform confirmed a marked increase in the percentage of oxidative fibres in the SOL and TA muscles of *Klf9* ^mlc−/−^ mice (Figure S2M,N).

### 3.4 Muscle Klf9 Regulates Systemic Glucose and Lipid Metabolism

Our RNA‐seq data revealed that the overexpression of Klf9 in skeletal muscle impaired metabolic pathways (Figure S1J). We next studied the metabolic phenotype of skeletal muscle‐specific *Klf9* transgenic and knockout mice. First, we measured the blood glucose levels of *Klf9* ^mlc−/−^ mice at 4 months of age fed a chow diet. Blood glucose levels in *Klf*9^mlc−/−^ mice fasted for 6 h were significantly lower than those in control mice (Figure S3A). Glucose tolerance tests (GTT) did not reveal a significant difference in glucose tolerance between the *Klf9* ^fl/fl^ and *Klf9* ^mlc−/−^ mice (Figure S3B). However, insulin tolerance tests (ITT) revealed increased insulin sensitivity in *Klf9* ^mlc−/−^ mice (Figure S3C). Consistently, the phosphorylation levels of AKT and rpS6 were increased in *Klf9* ^mlc−/−^ mice treated with or without insulin (Figure S3D).

We next fed *Klf9* ^mlc−/−^ mice and control mice a high‐fat diet (HFD) at 6 weeks of age. The body weights of these mice began to diverge at 9 weeks of age, with *Klf9* ^mlc−/−^ mice gaining less weight than control mice did despite having a similar food intake per day (Figure S3E–G). As observed in chow diet‐fed *Klf9* ^mlc−/−^ mice, the weight and size of skeletal muscle in high‐fat diet‐fed *Klf9* ^mlc−/−^ mice were also modestly and significantly increased. H&E staining confirmed the larger size of myofibers in the *Klf9* ^mlc−/−^ mice (Figure S3H–J). The weights and sizes of individual fat pads and the liver were markedly reduced in the *Klf9* ^mlc−/−^ mice (Figure S3K,L). Similarly, the sizes of the lipid droplets in the liver and adipocytes in the BAT and WAT of *Klf9* ^mlc−/−^ mice were smaller than those in control mice, as revealed by histological analysis (H&E staining) (Figure S3M,N). Consistent with the increased expression of genes involved in fatty acid oxidation and mitochondrial OXPHOS in skeletal muscle (Figure S3O), *Klf9* ^mlc−/−^ mice presented decreased serum triglyceride and cholesterol levels; moreover, hepatic triglyceride and cholesterol contents were also reduced, as revealed by biochemical analysis (Figure S3P).

GTT and ITT results revealed that Klf9 deficiency improved glucose intolerance and enhanced insulin sensitivity when the mice were maintained on a high‐fat diet (Figure S3Q,R). Consistent with the ITT data, the effects of insulin on AKT signalling in the GAS, eWAT and liver were enhanced in *Klf9* ^mlc−/−^ mice (Figure S3S). Taken together, these findings indicate that Klf9 in skeletal muscle plays an important role in regulating systemic glucose and lipid metabolism.

We also studied the effects of *Klf9* transgene expression on systemic metabolism. m*Klf9*TG and control mice were fed a chow diet. The blood glucose levels in the transgenic mice fasted for 6 h were modestly but significantly greater than those in the control mice (Figure S3T). Although the skeletal muscle‐specific *Klf9* transgene did not markedly affect glucose tolerance, it impaired insulin sensitivity in mice on a chow diet, as revealed by the GTT and ITT results (Figure S3U,V). We also examined these mice under high‐fat diet conditions. However, Klf9 overexpression in skeletal muscle did not significantly affect the metabolic phenotype of the mice (data not shown).

### 3.5 KLF9 Directly Binds to and Activates the Promoters of Myostatin and MAFbx

**FIGURE 4:**
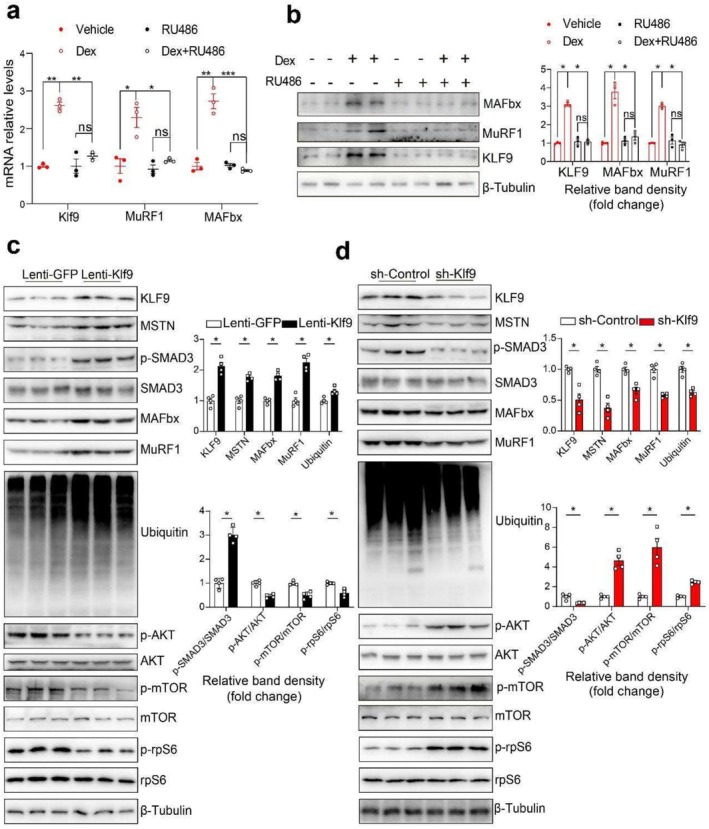
Klf9 overexpression activates muscle atrophy related genes of C2C12 myotubes, dependently on Mstn. (a) Quantitative PCR analysis of Klf9, MuRF1 and MAFbx mRNA levels in C2C12 myotubes treated with 10‐μM Dex and/or 10‐μM of the GR antagonist RU486 for 12 h (*n* = 3/group). (b) Representative Western blotting analysis of protein levels of KLF9, MuRF1 and MAFbx in C2C12 myotubes in (a) (left). Quantification of the KLF9/Tubulin, MuRF1/Tubulin and MAFbx/Tubulin signal ratios (right) (*n* = 3/group). (c) Representative Western blotting analysis of KLF9, MSTN, MuRF1, MAFbx, Ubiquitin and the phosphorylation level of SMAD3, AKT, mTOR and rpS6 protein levels from myotubes infected with lenti‐GFP or lenti‐*Klf9* lentivirus (left). Quantification of the KLF9/Tubulin, MSTN/Tubulin, MuRF1/Tubulin, MAFbx/Tubulin, Ubiquitin/Tubulin, p‐RPS6/RPS6, p‐AKT/AKT, p‐mTOR/mTOR and p‐SMAD3/SMAD3 signal ratios (right) (*n* = 4/group). (d) Representative Western blotting analysis of KLF9, MSTN, MuRF1, MAFbx, Ubiquitin and the phosphorylation level of SMAD3, AKT, mTOR and rpS6 protein levels from myotubes infected with sh‐Control or sh‐*Klf9* lentivirus (left). Quantification of the KLF9/Tubulin, MSTN/Tubulin, MuRF1/Tubulin, MAFbx/Tubulin, Ubiquitin/Tubulin, p‐RPS6/RPS6, p‐AKT/AKT, p‐mTOR/mTOR and p‐SMAD3/SMAD3 signal ratios (right) (*n* = 4/group). All data are shown as mean ± SEM. Unpaired two‐tailed Student's *t* tests were performed in (c) and (d) or two‐way ANOVAs were performed in (a) and (b).

Our data above revealed that skeletal muscle Klf9 expression was induced by GCs. GCs mediate physiological effects through binding to GR. Indeed, treatment with the GR antagonist RU486 almost completely abolished the Dex‐mediated induction of Klf9 and atrophy‐related genes in C2C12 myotubes (Figure 4a,b).

To explore whether the effects of Klf9 on muscle atrophy are cell autonomous, we constructed lentiviruses encoding *Klf9* (Lenti‐*Klf9*) and Lenti‐GFP (as a control) and performed Klf9 gain‐of‐function studies in differentiated C2C12 myotubes. The ectopic expression of Klf9 in C2C12 cells significantly increased the expression of skeletal muscle atrophy‐related genes and the content of ubiquitinated proteins in C2C12 cells. Meanwhile, the phosphorylation of signalling molecules controlling muscle protein synthesis, including AKT, mTOR and rpS6, was inhibited (Figures 4c and S4A). In contrast, Klf9 knockdown in differentiated C2C12 myotubes via Lenti‐sh*Klf9* (encoding a shRNA specific to *Klf9*) inhibited ubiquitin‐mediated protein degradation and activated protein synthesis (Figures 4d and S4B). Our above data suggest that Klf9 regulates myostatin expression in vivo and in vitro.

**FIGURE 5:**
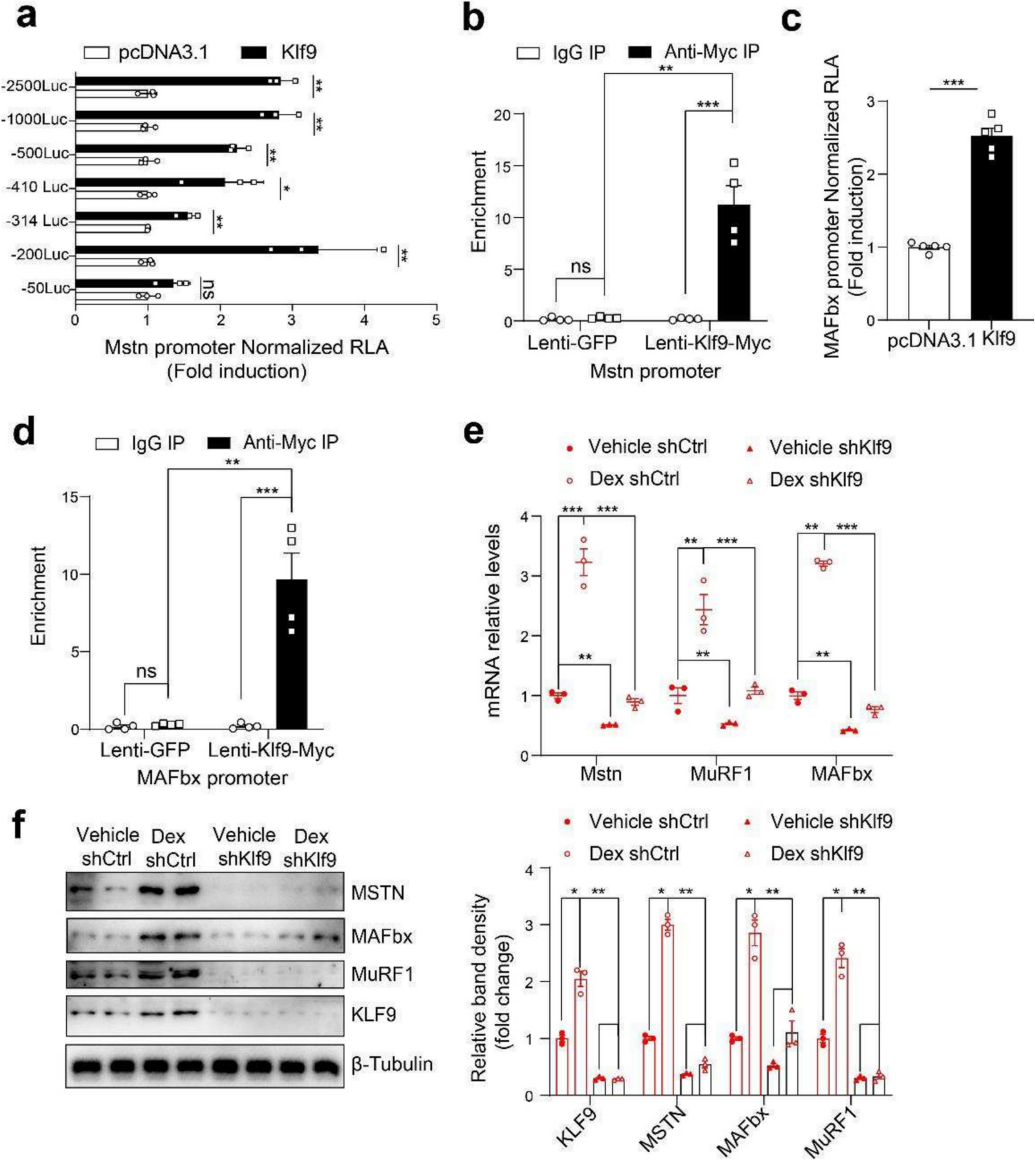
Klf9 induced atrophy by directly promoting myostatin and MAFbx expression. (a) Luciferase activity in C2C12 cells transfected with the indicated plasmids. A series of truncated Mstn promoters fused to the luciferase reporter gene were cotransfected into C2C12 cells, together with pcDNA3.1 (control, black bars) or Klf9‐expression plasmids (red bars) (*n* = 3/group). (b) ChIP assay was performed in C2C12 cells infected with lentivirus expressing Myc‐tagged Klf9 to assess Klf9 occupancy of the Mstn promoter. The Mstn promoter fragments containing the −200‐ to −50‐bp region could be amplified from the precipitates obtained with an anti‐Myc antibody but not with normal mouse IgG (control) (*n* = 4/group). (c) Luciferase activity in C2C12 cells transfected with the indicated plasmids. 3.3‐kb length promoters of MAFbx fused to the luciferase reporter gene were cotransfected into C2C12 cells, together with pcDNA3.1 (control, black bars) or Klf9‐expression plasmids (red bars) (*n* = 5/group). (d) ChIP assay was performed in C2C12 cells infected with lentivirus expressing Myc‐tagged KLF9 to assess KLF9 occupancy of the MAFbx promoter. The MAFbx promoter fragments containing the −3184‐ to −3090‐bp region could be amplified from the precipitates obtained with an anti‐Myc antibody but not with normal mouse IgG (control) (*n* = 4/group). (e) Quantitative PCR analysis of Mstn, MuRF1 and MAFbx mRNA levels in C2C12 myotubes treated with 10‐μM Dex or vehicle after treated with sh‐control and sh‐*Klf9* lentivirus (*n* = 3/group). (f) Representative Western blotting analysis of KLF9, MSTN, MuRF1 and MAFbx protein levels from C2C12 myotubes described in e (left). Quantification of the KLF9/Tubulin, MSTN/Tubulin, p‐MuRF1/Tubulin, MAFbx/Tubulin signal ratios (right) (*n* = 3/group). All data are shown as mean ± SEM. Unpaired two‐tailed Student's *t* tests were performed in (a) and (c) or one‐way ANOVAs were performed in (b) and (d) or two‐way ANOVAs were performed in (e) and (f).

We next tested the hypothesis that KLF9 directly activates Mstn gene transcription. A series of truncated segments of the promoter fused to the luciferase gene (−2500 Luc, −1000 Luc, −500 Luc, −410 Luc, −314 Luc and −200 Luc) were transfected into C2C12 myoblasts to map the cis‐acting element that confers the Klf9‐dependent activation of the reporter gene. The results revealed that the overexpression of KLF9 activated the transcription of the pGL3‐Mstn promoter regions at −2500, −1000, −500, −410, −314 and −200 bp in C2C12 myoblasts, whereas the stimulatory effects of Klf9 were abolished when the promoter region was further truncated to −50 bp, suggesting that a KLF9 binding site is present between −200 and −50 bp (Figure 5a). Additionally, the occupancy of KLF9 proteins on the Mstn promoter was confirmed by a ChIP assay using C2C12 myotubes (Figure 5b).

Similarly, we also observed that Klf9 overexpression in C2C12 cells activated the transcription of the pGL3‐MAFbx gene promoter (Figure 5c). Moreover, a ChIP assay using differentiated C2C12 myotubes infected with Lenti‐*Klf9* confirmed that the KLF9 protein binds to the MAFbx promoter region, which contains a potential KLF9 binding site (−3184 to −3090 bp) (Figure 5d). Taken together, these results suggest that KLF9 directly binds to and activates the transcription of the Mstn and MAFbx gene promoters.

We also found that *Klf9* knockdown reduced the Dex‐mediated induction of Mstn, MuRF1 and MAFbx expression in C2C12 myotubes (Figure 5e,f).

### 3.6 Myostatin Mediates the Effect of Klf9 on Muscle Mass and Function

Since myostatin is a master regulator of muscle mass and a direct downstream target of Klf9, we next explored whether the effects of Klf9 on muscle atrophy are myostatin dependent in vivo. We first generated an adeno‐associated virus encoding a protease‐resistant form of MSTN (AAV‐dominant negative MSTN, AAV2/8‐dnMstn) driven by the hepatocyte‐specific human thyroxine‐binding globulin (TBG) promoter (Figure S5A) to inhibit the activity of endogenous MSTN as previously reported [29, 30, 31]. AAV2/8‐dnMstn and AAV2/8‐GFP were injected into *Klf9* transgenic and control mice at 6 weeks of age via the tail vein. After 3 months, we observed that AAV2/8‐dnMstn infection markedly induced exogenous dominant negative MSTN expression in the livers of AAV2/8‐dnMstn‐injected mice (Figure S5B). Correspondingly, the phosphorylation of SMAD3 was reduced in the muscle of AAV2/8‐dnMstn‐injected mice, indicating that the activity of endogenous myostatin was significantly inhibited (Figure S5C). Consistently, the grip strength of *Klf9* transgenic mice improved after AAV2/8‐dnMstn infection (Figure S5D). The muscle masses of the GAS, TA, SOL and Quad muscles of the m*Klf9*TG mice were markedly greater than those of the AAV2/8‐dnMstn‐injected mice (Figure S5E). H&E staining of the TA muscle revealed that the myofiber size also increased after the inhibition of endogenous MSTN activity (Figure S5F).

**FIGURE 6:**
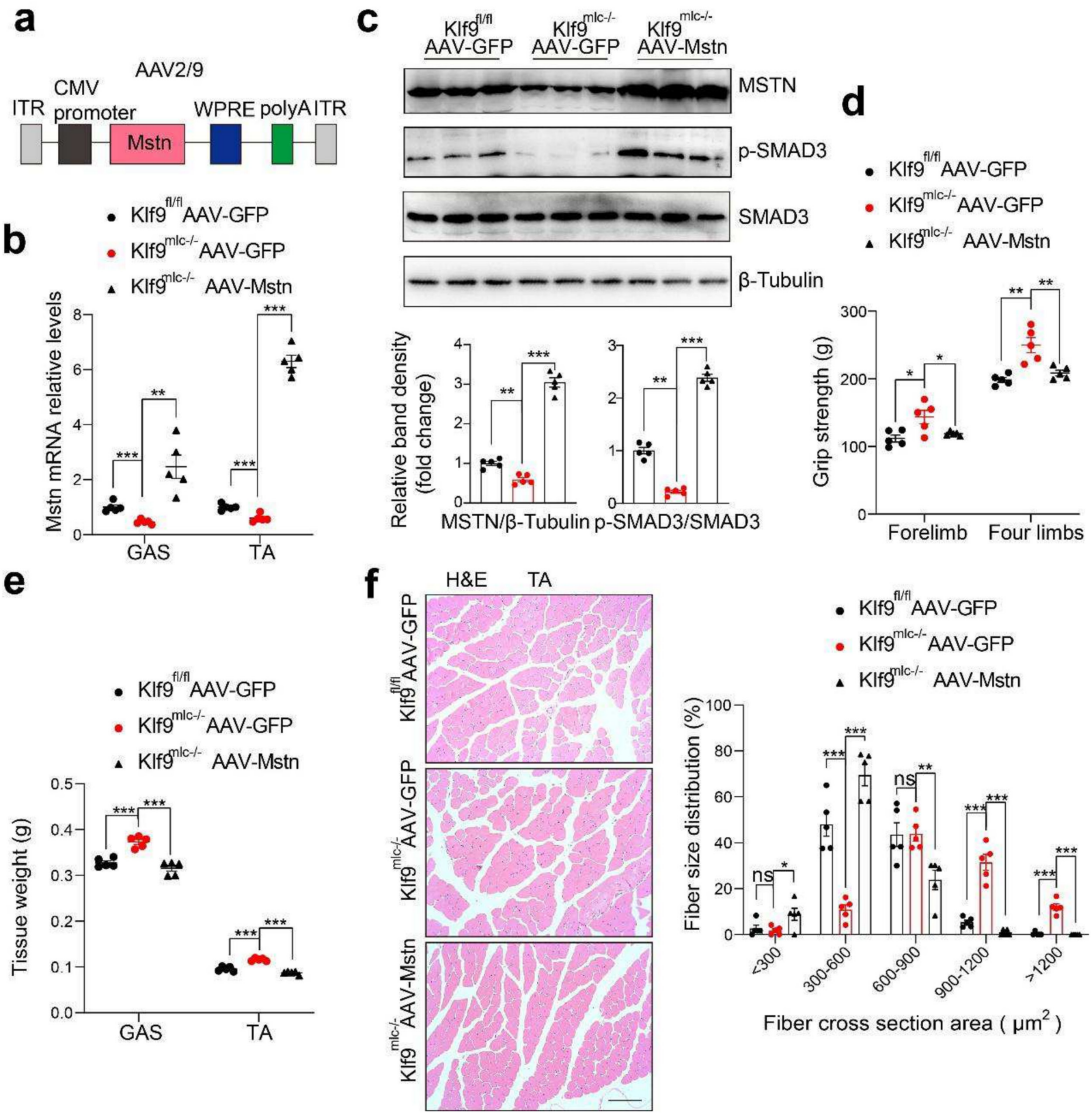
Regulation of muscle growth in *Klf9* ^mlc−/−^ mice by Klf9 via increase the amount of myostatin. (a) Schematic representation of the basic components of Mstn insert packaged inside recombinant AAV gene transfer vector. The vector was single‐stranded, contained ITR elements from AAV serotypes 2 and was packaged in serotype 9 capsids. (b) At 10 weeks of age, *Klf9* ^fl/fl^ and *Klf9* ^mlc−/−^ mice received a single adeno‐associated virus‐mediated GFP (AAV‐GFP) as control or adeno‐associated virus–mediated myostatin (Mstn) (AAV‐Mstn) through intramuscular injection into GAS and TA, and the terminal endpoint was 16 weeks of age. Quantitative PCR analysis of mRNA levels of Mstn in the skeletal muscle of AAV treated *Klf9* ^*fl/fl*^ and *Klf9* ^*mlc−/−*^ mice at age of 16 weeks old (*n* = 5/group). (c) Representative Western blotting analysis of MSTN, phosphorylation of SMAD3 in skeletal muscle from mice described in (a) (top). Quantification of the MSTN/Tubulin, SMAD3/SMAD3 signal ratios (bottom) (*n* = 5/group). (d) Forelimb and four‐limbs grip strength was tested in mice described in (a) (*n* = 5/group). (e) Tissue weights of GAS and TA from mice described in (a) (*n* = 5/group). (f) Haematoxylin and eosin staining of paraffin‐embedded TA sections of mice described in (a) (left). Quantification of the myofiber cross section area size of TA of the mice in (a) using Image J software (right) (*n* = 5/group). All data are shown as mean ± SEM. One‐way ANOVA was performed in (c) or two‐way ANOVAs were performed in (b), (d) and (e). Scale bars: 50 μm.

To further confirm whether myostatin mediates the physiological function of Klf9, we performed myostatin rescue experiments in *Klf9* ^mlc−/−^ mice. An AAV expressing WT MSTN (AAV2/9‐Mstn) driven by the CMV promoter was constructed and delivered to the GAS and TA muscles of *Klf9* ^mlc−/−^ and *Klf9* ^fl/fl^ mice via intramuscular injection (Figure 6a). Quantitative PCR and Western blotting revealed that AAV2/9‐Mstn mediated Mstn overexpression in the muscle of both *Klf9* ^mlc−/−^ and *Klf9* ^fl/fl^ mice (Figure 6b,c). Consistently, Mstn rescued the increased phosphorylation of SMAD3 in the muscle of *Klf9* ^mlc−/−^ mice (Figure 6c). Compared with AAV‐GFP‐infected *Klf9* ^mlc−/−^ mice, AAV‐Mstn‐infected *Klf9* ^mlc−/−^ mice had less grip strength (Figure 6d). In addition, the GAS and TA muscle tissue weights of AAV‐Mstn‐infected *Klf9* ^mlc−/−^ mice were lower than those of AAV‐GFP‐infected *Klf9* ^mlc−/−^ mice (Figure 6e). The myofiber size was smaller in the AAV‐Mstn‐infected *Klf9* ^mlc−/−^ mice than in the AAV‐GFP‐infected *Klf9* ^mlc−/−^ mice (Figure 6f). Thus, these data suggest that myostatin, at least in part, mediated the atrophic effects of the *Klf9* transgene.

### 3.7 Skeletal Muscle Klf9 Deficiency Alleviates Muscle Atrophy Induced by Short‐Term Acute Dex Treatment

**FIGURE 7:**
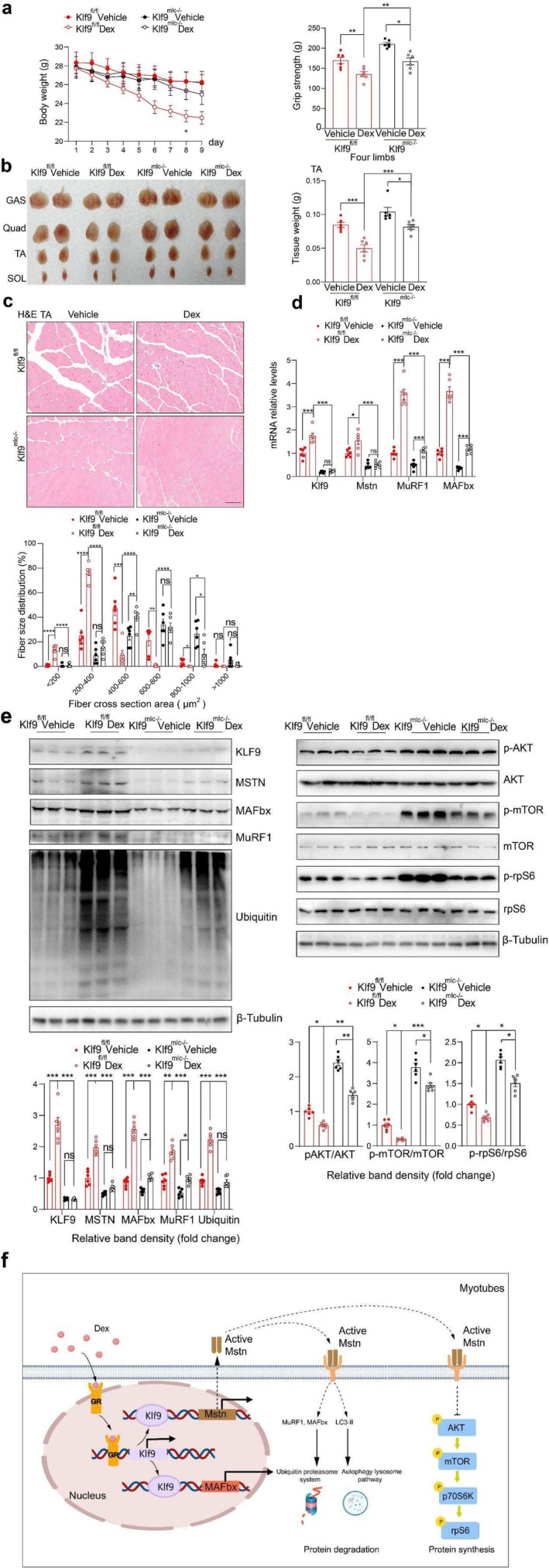
Skeletal muscle Klf9 deficiency alleviates muscle atrophy induced by short‐term acute Dex treatment. (a) Body weight of 10‐week old *Klf9* ^*fl/fl*^ and *Klf9* ^*mlc−/−*^ mice intraperitoneally treated with acute high doses of Dex (25 mg/kg/day) for 9 days (*n* = 6/group) (left). Grip strength of the indicated mice (right) (*n* = 6/group) (right). (b) Gross appearance of soleus (SOL), gastrocnemius (GAS), quadriceps (QUA) and tibialis anterior (TA) from *Klf9* ^*fl/fl*^ and *Klf9* ^*mlc−/−*^ mice treated with Dex (*n* = 6/group). Tissue weight of TA in mice described in (a) (*n* = 6/group). (c) Haematoxylin and eosin staining of paraffin‐embedded TA sections of mice described in (a) (top). Quantification of the myofiber cross section area size of TA of the mice in (a) using Image J software (bottom) (*n* = 6/group). (d) Quantitative PCR analysis of mRNA levels of Klf9 and atrophy related genes from muscles of *Klf9* ^*fl/fl*^ and *Klf9* ^*mlc−/−*^ mice in (a) (*n* = 6/group). (e) Representative Western blotting analysis of KLF9, MSTN, MuRF1, MAFbx and ubiquitin levels (top left) and the phosphorylation level of AKT, mTOR and rpS6 protein levels (top right) from mice described in (a) (left). Quantification of the KLF9/Tubulin, MSTN/Tubulin, MuRF1/Tubulin, MAFbx/Tubulin, Ubiquitin/Tubulin (bottom left), p‐RPS6/RPS6, p‐AKT/AKT and p‐mTOR/mTOR signal ratios (bottom right) (*n* = 6/group). (f) The proposed model of Klf9 regulating skeletal muscle mass. All data are shown as mean ± SEM. Two‐way ANOVAs were performed in (a)–(e).

Since GCs induce Klf9 expression in skeletal muscle, we next explored whether Klf9 alters the effects of GCs on muscle mass and function. We established a mouse model of GC‐induced muscle wasting via the exogenous administration of a high dose of Dex (25 mg/kg/day) or vehicle (control) for 9 days. *Klf9* ^fl/fl^ and *Klf9* ^mlc−/−^ mice were intraperitoneally injected with Dex. Compared with vehicle‐treated control mice, Dex‐treated *Klf9* ^fl/fl^ mice presented a gradual decrease in body weight and attenuated four limbs grip strength after Dex administration. However, these effects of Dex were abolished in *Klf9* ^mlc−/−^ mice (Figure 7a). Consistent with the above data, skeletal muscle‐specific *Klf9* deficiency increased muscle mass and myofiber size. Dex treatment markedly reduced muscle mass and myofiber size in *Klf9* ^fl/fl^ mice. Notably, although Dex still reduced the muscle mass and size in *Klf9* ^mlc−/−^ mice, the magnitude of the reduction was significantly lower than that in *Klf9* ^fl/fl^ mice (Figure 7b,c). Consistently, skeletal muscle *Klf9* deficiency significantly reduced the acute high‐dose Dex‐mediated induction of atrophy‐related gene expression (Figure 7d,e).

On the basis of these data, we propose a model of GC‐mediated induction of skeletal muscle atrophy. The GC/GR complex induces Klf9 expression in muscle, which in turn activates the transcription of Mstn and MAFbx, thereby increasing skeletal muscle protein degradation and inhibiting skeletal muscle protein synthesis, subsequently causing muscle atrophy. Our study provides novel insights into the GC‐induced induction of atrophy. Thus, targeting Klf9 might have therapeutic potential in the treatment of muscle loss diseases (Figure 7f).

## 4 Discussion

Skeletal muscle mass maintenance is important for physical activity and whole‐body metabolism. In this study, we revealed the physiological function of Klf9 in skeletal muscle. Compared with control mice, mice with skeletal muscle‐specific *Klf9* overexpression presented sharply decreased muscle mass and impaired muscle function. The size and weight of skeletal muscle tissue were decreased in m*Klf9*TG mice. Consistently, skeletal muscle strength and running ability were also markedly impaired in these *Klf9* transgenic mice. Conversely, Klf9 deficiency in skeletal muscle resulted in increased muscle mass and increased muscle strength. Moreover, Klf9 deficiency in mice alleviated high‐fat diet‐induced obesity and improved glucose intolerance and insulin resistance. Mechanistically, Klf9 promoted the expression of atrophy‐linked ubiquitin ligases, including MuRF1, MAFbx and myostatin, and inhibited the AKT/mTOR/p70S6K signalling pathway, which controls protein synthesis.

Skeletal muscle contains myofibers that differ in contractile function, mitochondrial content and metabolic characteristics. Type I (slow‐twitch) myofibers contain a high concentration of mitochondria and use oxidative phosphorylation as the main energy source. However, Type II (fast‐twitch) myofibers generate ATP via glycolysis [32]. GCs have been shown to cause fast‐twitch muscle fibre atrophy, with less or no impact observed in slow‐twitch fibres [6]. An explanation for this difference in atrophy is the differential expression of GR between fibre types. Fast‐twitch muscle expresses abundant GR, whereas slow‐twitch muscle expresses low levels of GR [33]. In the present study, we show that Dex‐inducible Klf9 controls skeletal muscle mass and function, and Klf9 overexpression in muscles led to a decreased proportion of oxidative Types I and IIa myofibers.

Myostatin is a negative regulator of muscle mass, and its inhibition has been proposed as a promising therapeutic strategy for muscle wasting. Myostatin is known to substantially increase muscle protein degradation via the MuRF1‐ or atrogin‐1‐mediated ubiquitin‐proteasome system [34]. However, blocking endogenous MSTN via AAV‐mediated dominant‐negative MSTN (dnMstn) overexpression partially, rather than completely, restored muscle mass and function in m*Klf9*TG mice to those of control mice. We also identified MAFbx as a direct target gene of Klf9. Therefore, ChIP‐seq of skeletal muscle from m*Klf9*TG mice is necessary to identify other downstream target genes of Klf9.

Myostatin (Mstn) is also expressed in brown fat [10]. We have previously demonstrated that Klf9 regulates energy metabolism of brown and beige fat [16]. However, it appears that the Klf9 transgene or deficiency in adipocytes does not alter the expression level of Mstn in brown fat (data not shown). Thus, Klf9 regulates Mstn expression in a tissue‐specific manner.

In the current study, we showed that Klf9 overexpression in skeletal muscle impaired mitochondrial function and suppressed fatty acid oxidation in mice. Conversely, Klf9 deficiency in skeletal muscle led to increased mitochondrial content, enhanced mitochondrial function and improved fatty acid oxidation. However, the exact mechanism underlying Klf9 regulating the expression of these genes related to energy metabolism remains unknown. Although Klf9 controls Mstn expression in muscle, the physiological role of myostatin in regulating mitochondrial energy metabolism remains controversial. Zhang et al. reported that myostatin deficiency increased fatty acid oxidation in muscle and other peripheral tissues [35]. However, other groups reported that the loss of myostatin impaired mitochondrial energy metabolism [36, 37]. KLF9 was reported to contain three C2H2 zinc finger motifs in the C‐terminal region and a SIN3A‐binding motif in the N‐terminal region [38]. Furthermore, SIN3A recruits HDACs to form the SIN3A/HDAC complex, thereby inhibiting histone acetylation levels and downstream gene expression [39]. Thus, we speculated that Klf9 exerts its effects via recruiting the SIN3A/HDAC complex to promoters of these genes. The precise regulatory mechanisms require further investigation.

Muscle regeneration relies on the activation and expansion of satellite cells, followed by myoblast differentiation and fusion into multinucleated myotubes. Then, myotubes undergo hypertrophic remodelling to generate muscle fibres [40]. However, whether Klf9 affects satellite cell activation and myoblast differentiation remains unexplored. It is necessary to generate a satellite cell‐specific *Klf9* knockout mouse model to clarify this issue.

In the present study, we show that Klf9 is induced by GC treatment. An acute and high‐dose GC‐induced muscle wasting mouse model was used to assess whether Klf9 alters the effects of Dex on atrophy. Skeletal muscle Klf9 deficiency alleviated the muscle atrophy induced by Dex rather than completely blocking the actions of Dex. These findings indicate that Klf9 partially mediates the Dex effect and that other factors are involved in this process. In fact, Shimizu et al. reported that GCs also induce the expression of Krüppel‐like transcription factor 15 (Klf15), another member of the Krüppel‐like factor family, which in turn regulates the expression of MAFbx and MuRF1 [33]. KLF15 interacts with the promoter regions of both MAFbx and MuRF1 to induce their expression. Thus, both of these factors may mediate the effect of Dex on atrophy. These studies highlight the complexity of the mechanisms responsible for GC‐induced muscle atrophy.

In summary, our results revealed a crucial role of Klf9 in regulating skeletal muscle mass. The targeting of skeletal muscle Klf9 may represent a potential avenue for therapeutic interventions for high‐dose GC‐induced skeletal muscle mass loss‐related diseases.

## Ethics Statement

All animal studies were conducted in strict accordance with the institutional guidelines for the humane treatment of animals and were approved by the IACUC committees of Tianjin medical university (Approval No. TMUaMEC2021052). All mice used in this study were housed and maintained in 12‐h light and dark photoperiods.

## Conflicts of Interest

The authors declare no conflicts of interest.

## Supporting information


**Data S1** Supplementary Information.

**Table S1** List of specific primers used for real‐time quantitative PCR gene expression analysis and ChIP analysis.

**Figure S1** **Generation and characterization of skeletal muscle‐specific *Klf9* transgenic mice.** (**a**) Generation of skeletal muscle‐specific Klf9‐overexpression mice. *Rosa26*‐LSL‐*Klf9* mice were generated using the CRISPR/Cas9 system to insert the CAG‐LoxP‐STOP‐LoxP‐Klf9 cassette into the mouse *Rosa26* locus. These mice were subsequently bred to MLC‐Cre transgenic mice to obtain m*Klf9*TG mice, leading to skeletal muscle‐specific Klf9 overexpression within the skeletal muscle. (**b**) Quantitative PCR analysis of mRNA levels of Klf9 in skeletal muscle and heart from R‐loxP and m*Klf9*TG mice at age of 3 months (*n* = 6/group). (**c**) Representative Western blotting analysis of KLF9 in skeletal muscle and heart from 3‐month old R‐loxP and m*Klf9*TG mice. **Quantification of the KLF9/Tubulin signal ratios (upper) (*n* = 6/group).** (**d**) Daily food intake of R‐loxP and m*Klf9*TG mice (*n* = 6/group). (**e**) Skeletal muscle tissue weight of R‐loxP and *Klf9*TG mice (*n* = 6/group). (**f**) Tissue weight of Liver, BAT, IW and EW from R‐loxP and m*Klf9*TG mice (*n* = 6/group). (**g**) Quantification of the myofiber cross section area size of TA from R‐loxP and m*Klf9*TG mice using Image J software (*n* = 6/group). (**h**) Volcano plot of differentially expressed genes in skeletal muscles of R‐loxP and m*Klf9*TG mice (Down: *p* < 0.01 and log2FC < −2; Up: p < 0.01 and log2FC > 2). KEGG analysis of up‐regulated (**i**) and down‐regulated (**j**) differentially expressed genes in skeletal muscle from R‐loxP and m*Klf9*TG mice. (**k**) Heatmap of differentially expressed genes in skeletal muscle of R‐loxP and m*Klf9*TG mice. (**l**) **Quantitative PCR analysis of mRNA levels of Klf9, Mstn, MuRF1 and MAFbx in skeletal muscle from** R‐loxP and m*Klf9*TG **mice (*n* = 6/group). (m) Western blotting analysis of GAS muscle total protein extracts prepared from indicated mice treated for 24 h with or without Colchicine (Col) (left). Quantification of LC3‐II/LC3‐I (right) (*n* = 6/group). (n)** Quantitative PCR analysis of mRNA levels of fatty acid oxidation and mitochondrial biogenesis related genes in skeletal muscle from R‐loxP and m*Klf9*TG mice at age of 3 months (*n* = 6/group). (**o**) Relative mtDNA levels of skeletal muscle from R‐loxP and m*Klf9*TG mice at age of 3 months (*n* = 6/group). (**p**) Representative electron micrographs of the soleus muscle showing mitochondria in sections from 12‐week‐old R‐loxP and m*Klf9*TG mice (*n* = 6 mice/group). The left scale bar represents 2 μm and the right scale bar represents 0.5 μm. **(q) Representative image (left) and quantification (right) of the NADH‐TR staining of the SOL and TA muscles of R‐loxP and m*Klf9*TG mice (*n* = 6/group). (r) Relative mRNA levels of type I, IIA, IIX or IIB muscle markers (myosin heavy chain [Myh] 7, Myh2, Myh1 and Myh4) in the SOL (top) and TA (bottom) muscles of R‐loxP and m*Klf9*TG mice (*n* = 6/group). All data are shown as mean ± SEM unpaired two‐tailed Student's t tests were performed in (b, d‐g, l, n, o, q and r). One‐way ANOVA was performed in m. Scale bars: 20 μm**.
**Figure S2. Generation and characterization of skeletal muscle‐specific Klf9‐knockout mice.** (**a**) Generation of skeletal muscle‐specific Klf9‐knockout (*Klf9* ^mlc−/−^) mice. *Klf9* ^fl/fl^ mice were generated by the CRISPR/Cas9 system to insert two loxP sites into exon1 of the Klf9 gene. These mice were subsequently bred with MLC‐Cre transgenic mice to obtain *Klf9* ^mlc−/−^ mice. (**b**) Quantitative PCR analysis of mRNA levels of Klf9 in skeletal muscle and heart from *Klf9* ^fl/fl^ and *Klf9* ^mlc−/−^ mice at age of 4 months (*n* = 6/group). (**c**) Representative Western blotting analysis of KLF9 in skeletal muscle and heart from *Klf9* ^fl/fl^ and *Klf9* ^*mlc−/−*^ mice at age of 4 months. **Quantification of the KLF9/Tubulin signal ratios (upper) (*n* = 6/group).** (**d**) Daily food intake of *Klf9* ^fl/fl^ and *Klf9* ^mlc−/−^ mice (*n* = 6/group). (**e**) Skeletal muscle tissue weight of *Klf9* ^fl/fl^ and *Klf9* ^mlc−/−^ mice (right) (*n* = 6/group). (**f**) Tissue weight of Liver, BAT, IW and EW from *Klf9* ^fl/fl^ and *Klf9* ^mlc−/−^ mice (*n* = 6/group). (**g**) Quantification of the myofiber cross section area size of TA from *Klf9* ^fl/fl^ and *Klf9* ^mlc−/−^ mice using Image J software (*n* = 6/group). (**h**) **Quantitative PCR analysis of mRNA levels of Klf9, Mstn, MuRF1 and MAFbx in skeletal muscle from** *Klf9* ^fl/fl^ and *Klf9* ^mlc−/−^ **mice (*n* = 6/group). (i) Western blotting analysis of GAS muscle total protein extracts prepared from indicated mice treated for 24 h with or without Colchicine (Col) (top). Quantification of LC3‐II/LC3‐I (bottom) (*n* = 6/group). (j)** Quantitative PCR analysis of mRNA levels of fatty acid oxidation and mitochondrial biogenesis related genes in skeletal muscle from *Klf9* ^fl/fl^ and *Klf9* ^mlc−/−^ mice at age of 4 months (*n* = 6/group). (**k**) Relative mtDNA levels of skeletal muscle from *Klf9* ^fl/fl^ and *Klf9* ^mlc−/−^ mice at age of 4 months (*n* = 6/group). (**l**) Representative electron micrographs of the soleus muscle showing mitochondria in sections from 4 month‐old *Klf9* ^fl/fl^ and *Klf9* ^mlc−/−^ mice (*n* = 6/group). The left scale bar represents 2 μm and the right scale bar represents 0.5 μm. **(m) Representative image (left) and quantification (right) of the NADH‐TR staining of the SOL and TA muscles of *Klf9*** ^**fl/fl**^ **and *Klf9*** ^**mlc−/−**^ **mice (*n* = 6/group). (n) Relative mRNA levels of type I, IIA, IIX or IIB muscle markers (myosin heavy chain [Myh] 7, Myh2, Myh1 and Myh4) in the SOL (left) and TA (right) muscles of *Klf9*** ^**fl/fl**^ **and *Klf9*** ^**mlc−/−**^ **mice (*n* = 6/group). All data are shown as mean ± SEM. unpaired two‐tailed Student's t tests were performed in (b, d‐h, j, k, m and n). One‐way ANOVA was performed in i. Scale bars: 20 μm**.
**Figure S3. Muscle Klf9 regulates systemic glucose and lipid metabolism.** Blood glucose levels of 6 h‐fasted *Klf9* ^fl/fl^ and *Klf9* ^mlc−/−^ mice at age of 4 months (*n* = 6/group). (**b**, **c**) Blood glucose levels during GTT (**b**) and ITT (**c**) performed in the mice in **a** (*n* = 6/group). (**d**) Western blotting analysis of AKT and p‐AKT (Ser473), rpS6 and p‐rpS6 in the skeletal muscle and 15 min after administration of insulin (1 U/kg) of indicated mice. **Quantification of the p‐AKT/AKT signal ratios (upper) (*n* = 6/group)**. (**e**) The growth curve of *Klf9* ^fl/fl^ and *Klf9* ^mlc−/−^ mice fed a HFD starting at 6 weeks of age (*n* = 6/group). (**f**) Gross morphology of *Klf9* ^fl/fl^ and *Klf9* ^mlc−/−^ mice fed a HFD for 2 months. (**g**) Daily food intake of *Klf9* ^fl/fl^ and *Klf9* ^mlc−/−^ mice fed a HFD for 2 months **(*n* = 6/group)**. (**h**) Gross appearance of soleus (SOL), gastrocnemius (GAS), quadriceps (QUA) and tibialis anterior (TA) from *Klf9* ^fl/fl^ and *Klf9* ^mlc−/−^ mice described in **b**. (**i**) Tissue weight of soleus (SOL), gastrocnemius (GAS), quadriceps (QUA) and tibialis anterior (TA) from the indicated mice (*n* = 6/group). (**j**) Haematoxylin and eosin staining of paraffin‐embedded TA pad sections from the mice in **h**. (**k**) Gross appearance of liver, interscapular BAT, inguinal and epididymal fat pads from mice in **h**. (**l**) Tissue weight of liver, interscapular BAT, inguinal and epididymal fat pads from mice in **h** (*n* = 6/group). (**m**) Haematoxylin and eosin staining and Oil Red O staining of paraffin‐embedded liver from the mice in **h**. (**n**) Haematoxylin and eosin staining of paraffin‐embedded BAT, inguinal and epididymal fat pad sections from the mice in **h**. (**o**) Quantitative PCR analysis of Klf9, Mstn, fatty acid oxidation and mitochondrial oxidative phosphorylation genes of GAS of the mice in **h** (*n* = 6/group). (**p**) Hepatic triglyceride, cholesterol, serum concentrations of triglyceride and cholesterol of mice described in **h** (*n* = 6/group). (**q**, **r**) Blood glucose levels during GTT (**q**) and ITT (**r**) performed in the mice in **h** (*n* = 6/group). (**s**) Western blotting analysis of AKT and p‐AKT (Ser473) in the skeletal muscle, adipose tissue and liver and 15 min after administration of insulin **(left)** (1 U/kg). **Quantification of the p‐AKT/AKT signal ratios (right) (*n* = 6/group).** (**t**) Blood glucose levels of 6 h‐fasted mice in R‐loxP and mKlf9TG mice at age of 3 months (*n* = 6/group). (**u**, **v**) Blood glucose levels during GTT (**u**) and ITT (**v**) performed in the mice in **s** (*n* = 6/group). All data are shown as mean ± SEM. unpaired two‐tailed Student's t tests were performed in (**a**‐**c**, **e**, **g**, **i**, **l**, **o**‐**r** and **t**‐**v**). **Two‐way ANOVA was performed in s**. Scale bars: 50 μm.
**Figure S4.** (Supplementary to Figure 4). (a) Quantitative PCR analysis of mRNA levels of Klf9 and atrophy related genes from myotubes treated with Lenti‐control and Lenti‐*Klf9* lentivirus (*n* = 4/group). (b) Quantitative PCR analysis of mRNA levels of Klf9 and atrophy related genes from myotubes treated with sh‐control and sh‐*Klf9* lentivirus (*n* = 4/group). All data are shown as mean ± SEM. Unpaired two‐tailed Student's t tests were performed in (a‐b).
**Figure S5. Regulation of muscle growth in m*Klf9*TG mice via inhibition the activity of myostatin.** (**a**) Schematic representation of the basic components of dnMstn insert packaged inside recombinant AAV gene transfer vector. The vector was single‐stranded, contained ITR elements from AAV serotypes 2, and was packaged in serotype 8 capsids. (**b**) At 6 weeks of age, R‐loxP and m*Klf9*TG mice received a single adeno‐associated virus–mediated GFP (AAV‐GFP) as control or adeno‐associated virus–mediated myostatin (Mstn) inhibitor (AAV‐dnMstn) through i.v. injection, and the terminal endpoint was 18 weeks of age (*n* = 5/group). Quantitative PCR analysis of mRNA levels of dnMstn in the liver of AAV treated R‐loxP and m*Klf9*TG mice at age of 18 weeks old (*n* = 5/group). (**c**) Representative Western blotting analysis of phosphorylation of SMAD3 in skeletal muscle from mice described in **a (top)**. **Quantification of the SMAD3/SMAD3 signal ratios (bottom) (*n* = 5/group)**. (**d**) Forelimb and four‐limbs grip strength was tested in mice described in **a** (*n* = 5/group). (**e**) Skeletal muscle tissue weight of mice described in **a** (*n* = 5/group). (**f**) Haematoxylin and eosin staining of paraffin‐embedded TA sections of mice described in **a** (left). Quantification of the myofiber cross section area size of TA of the mice in **a** using Image J software (right) (*n* = 5/group). **All data are shown as mean ± SEM. One‐way ANOVAs were performed in b and c or two‐way ANOVAs were performed in (d—f)**. Scale bars: 50 μm.

## Data Availability

The paper and the supporting information present all data needed to evaluate the conclusions. Datasets used and/or analysed during the current study are available from the corresponding author on reasonable request.
