# Supplementary material for: Klf9 Loss of Function Protects Against Glucocorticoids Induced Skeletal Muscle Wasting
Source: J Cachexia Sarcopenia Muscle. 2025 Jul 28;16(4):e70020. doi: 10.1002/jcsm.70020 (PMC12301625; doi:10.1002/jcsm.70020)
Supplement: Supplementary file 1 — Data S1 Supplementary Information. [file JCSM-16-e70020-s001.docx]

**Supplementary Material**

**Klf9 loss of function protects against glucocorticoids induced skeletal muscle wasting**

Yujie Zhang, Jingran Hao, Yueyao Feng, Tongtong Qiu, Jinjin Wu, Xuenan Zhou, Heng Fan, Yongsheng Chang

Correspondence: Yongsheng Chang, Tianjin 300070, China. [changys@tmu.edu.cn](mailto:changys@tmu.edu.cn)

**Additional supplementary materials and methods**

**Additional animal studies**

For DIO studies, male 4-week-old mice were fed on HFD (D12492; Research Diets) for 3 months.

For acute muscle wasting models, male 12-week *Klf9^fl/fl^* and *Klf9^mlc-/-^* mice were intraperitoneally injected with 25 mg/kg/day Dexamethasone (MCE; HY-14648) for 9 days.

Body weight was measured weekly. Body composition (fat and lean mass) was determined by MRI (Echomri, Combo-700).

HLS Model: The HLS method was performed as previous described. Briefly, 10-week old male C57BL/6J WT mice were HLS for 10 days. The reloaded mice were suspension for 10 days and then reloaded for another 4 days. The weight of skeletal muscle was measured after the mice were sacrificed.

Exercise Capacity Measurement: 3-month old mice of m*Klf9*TG and 4-month old mice of *Klf9^mlc-/-^* with their control littermates were acclimated to the treadmill 4-5 days prior to the exercise test session. Before each session, food was removed 2 hr before exercise. Acclimation began at a low speed of 5 to 8 meters per minute (m/min) for a total of 10 min on Day 1, and was increased to 5 to 10 m/min for a total of 10 min on Day 2. Following this, the mice were allowed to rest for at least 2 days in their home cage. On the day of test, the treadmill began at a rate of 12 m/min for 40 min. After 40 min, the treadmill speed was increased at a rate of 1 m/min every 10 min for a total of 30 min, and then increased at the rate of 1 m/min every 5 min until the mice were exhausted (mice spent more than 5 seconds on the electric shocker without resuming running). Individual experiments were replicated 2-4 times.

**AAV construction**

dnMstn AAV construction: The myostatin gene was isolated from mouse cDNA and the C terminal region from nucleotide 825–1131 of the reading frame was deleted via PCR. Splicing by overlap extension PCR was then used to introduce a D76A mutation that results in a peptide resistant to proteolytic activation, as previously reported with some modifies.^1-3^ The resulting mutant transgene was named dominant negative myostatin (dnMstn) and was cloned into an AAV transfer vector with a liver specific promoter (TBG), AAV-TBG-GFP (Addgene, 105535) and was co-transfected with AAV pseudotype 2/8 (Addgene, 112864) and pAdDeltaF6 (Addgene, 112867) into 293T cell line to produce virus. AAV-TBG-GFP was co- transfected with AAV pseudotype 2/8 and pAdDeltaF6 into 293T cell line to produce control virus. And virus was purified using Iodixanol. 2*10^12^ vg/mice AAV were injected into indicated mice via tail vein injection.

AAV-Mstn construction: The full length myostatin gene was isolated from mouse cDNA and inserted into the AAV2/9-CMV plasmid. AAV2/9-Mstn, AAV pseudotype 9 and pAdDeltaF6 were co-transfected into 293T cell line. AAV2/9-CMV plasmid was co-transfected with AAV pseudotype 9 and pAdDeltaF6 to produce control virus. AAV production was conducted as above. 1.5*10^12^ vg/mice AAV were injected into the GAS and TA muscle of indicated mice.

**In vivo protein synthesis rate measurement**

In vivo protein synthesis rates were measured via the SUnSET technique. Three-month old male R-loxP and m*Klf9*TG mice were intraperitoneal injected with 0.04 μmol puromycin/g mice. Thirty minute post-injection, muscle were collected and frozen in liquid Nitrogen for western blotting analysis using anti-puromycin antibody (A23031, Abclonal).

**Glucose and insulin tolerance test**

Glucose and insulin tolerance tests were performed as described.^4^ For GTT, mice fasted overnight were injected intraperitoneally with D-glucose (2 g/kg). For ITT, mice fasted for 6 hours were injected intraperitoneally with human insulin (Sigma) (0.75 U/kg). Blood glucose concentrations were measured from the tail vein at indicated times.

**Immunoblotting analysis**

Cell, skeletal muscle tissue and other tissues were lysed using RIPA lysis buffer (150mM NaCl, 1%Triton-x-100, 1%SDS, 50mM Tris-Cl pH8.0, 0.5% Deoxycholic acidsodium salt) supplemented with complete protease inhibitor cocktail (Roche) and PMSF (Roche) on ice. Immunoblotting was performed using the following primary antibodies: KLF9 (A7196; ABclonal); p-AKT (Ser473) (Cell Signaling Technology (CST), #9271); AKT (CST, #9272); S6 Ribosomal Protein (rpS6) (A11874; ABclonal); Phospho-S6 Ribosomal Protein (rpS6)-S235/236 Rabbit pAb (AP0538); MSTN Rabbit pAb (A6913); β-Tubulin (YM3030; Immunoway).

**RNA extraction and quantitative PCR analysis**

Total RNA was extracted from tissues or cells with TRIzol reagent (Solarbio). Real-time PCR was performed as described previously.^5^ The related primers are shown in Supplemental Table 1.

**Lentivirus Production and Purification**

The full-length mouse Klf9 gene containing a Myc-tag at C-terminus was cloned into pCDH-CMV-MCS-EF1-copGFP, and co-transfected with pMD2G and psPAX2 to HEK-293T cells. The concentrated viruses were used to infect differentiated C2C12 cells. For Klf9 knockdown, short-hairpin RNA (shRNA) sequences were synthesized by Tsingke Biotechnology Co., Ltd. (China) and constructed into lentivirus plasmids pLKO.1-TRC Cloning Vector (Addgene, No.10878). Lentiviruses were generated as previously described.^6^ The concentrated viruses were used to infect C2C12 cells.

**Construction of the reporter gene plasmids**

pGL3-Mstn promoter (−2500bp to +100bp) were constructed with KpnI/XhoI sites. A series of 5′ deletion constructs of Mstn promoter (−1000Luc, −500Luc, −410Luc, -314Luc, -200Luc, -50Luc) were prepared by PCR using −2500Luc as a template. Luciferase was measured as described previously ^5^.

**Transient transfection and luciferase assays**

The C2C12 cells were grown in 24-well plates using Dulbecco's Modified Eagle Medium (GIBCO/BRL, 37 °C, 5% CO2) containing 10% fetal bovine serum (FBS). The cells were co-transfected with each of the indicated luciferase reporter and expression plasmids using Lipofectamine TM 2000 (Invitrogen) according to manufacturer’s recommendations. Cotransfections were performed with a constant amount of DNA by adding the empty pcDNA3.1. A Renilla luciferase expression vector, pCMV-RL-TK, was used as an internal control to adjust for the transfection efficiency. The luciferase activity was measured at 48 hr after transfection using the Dual Luciferase Reporter Assay System (Promega). In vitro muscle wasting model was constructed in differentiated C2C12 myotubes with 10μM Dex for 12 hours.

**RNA sequencing**

For the RNA sequencing assay, 3-month old mice of m*Klf9*TG and their littermate control mice were sacrificed and total RNA was extracted from indicated muscle using TRIzol (Invitrogen) according to the manufacturer’s instructions. cDNA libraries were constructed, and single-end libraries were sequenced using the MGISEQ-2000 platform (BGI Genomics, Shenzhen). Data was analysed using the MegaBOLT platform (BGI Genomics, Shenzhen) and differentially expressed genes (DEGs) were screened using two criteria: (1) a fold change greater than 2 and (2) a corresponding adjusted P value less than 0.01.

**References**

1 Morine, K. J. *et al.* Systemic myostatin inhibition via liver-targeted gene transfer in normal and dystrophic mice. *PLoS One* **5**, e9176, doi:10.1371/journal.pone.0009176 (2010).

2 Liu, M., Hammers, D. W., Barton, E. R. & Sweeney, H. L. Activin Receptor Type IIB Inhibition Improves Muscle Phenotype and Function in a Mouse Model of Spinal Muscular Atrophy. *PLoS One* **11**, e0166803, doi:10.1371/journal.pone.0166803 (2016).

3 Hammers, D. W. *et al.* Glucocorticoids counteract hypertrophic effects of myostatin inhibition in dystrophic muscle. *JCI Insight* **5**, doi:10.1172/jci.insight.133276 (2020).

4 Fan, H. *et al.* Cold-Inducible Klf9 Regulates Thermogenesis of Brown and Beige Fat. *Diabetes* **69**, 2603-2618, doi:10.2337/db19-1153 (2020).

5 Cui, A. *et al.* Dexamethasone-induced Kruppel-like factor 9 expression promotes hepatic gluconeogenesis and hyperglycemia. *J Clin Invest* **129**, 2266-2278, doi:10.1172/JCI66062 (2019).

6 Yang, L., Zhou, Z. G. & Sun, X. F. Specific knockdown of PPARdelta gene in colon cancer cells by lentivirus-mediated RNA interfering. *Methods Mol Biol* **952**, 67-86, doi:10.1007/978-1-62703-155-4_5 (2013).

**Supplementary figure captions**

**Figure S1. Generation and characterization of skeletal muscle-specific *Klf9* transgenic mice**

(**a**) Generation of skeletal muscle-specific Klf9-overexpression mice. *Rosa26*-LSL-*Klf9* mice were generated using the CRISPR/Cas9 system to insert the CAG-LoxP-STOP-LoxP-Klf9 cassette into the mouse *Rosa26* locus. These mice were subsequently bred to MLC-Cre transgenic mice to obtain m*Klf9*TG mice, leading to skeletal muscle-specific Klf9 overexpression within the skeletal muscle. (**b**) Quantitative PCR analysis of mRNA levels of Klf9 in skeletal muscle and heart from R-loxP and m*Klf9*TG mice at age of 3 months (n = 6/group). (**c**) Representative Western blotting analysis of KLF9 in skeletal muscle and heart from 3-month old R-loxP and m*Klf9*TG mice. **Quantification of the KLF9/Tubulin signal ratios (upper) (n = 6/group).** (**d**) Daily food intake of R-loxP and m*Klf9*TG mice (n = 6/group). (**e**) Skeletal muscle tissue weight of R-loxP and *Klf9*TG mice (n = 6/group). (**f**) Tissue weight of Liver, BAT, IW and EW from R-loxP and m*Klf9*TG mice (n = 6/group). (**g**) Quantification of the myofiber cross section area size of TA from R-loxP and m*Klf9*TG mice using Image J software (n = 6/group). (**h**) Volcano plot of differentially expressed genes in skeletal muscles of R-loxP and m*Klf9*TG mice (Down: p < 0.01 and log2FC < -2; Up: p < 0.01 and log2FC > 2). KEGG analysis of up-regulated (**i**) and down-regulated (**j**) differentially expressed genes in skeletal muscle from R-loxP and m*Klf9*TG mice. (**k**) Heatmap of differentially expressed genes in skeletal muscle of R-loxP and m*Klf9*TG mice. (**l**) **Quantitative PCR analysis of mRNA levels of Klf9, Mstn, MuRF1 and MAFbx in skeletal muscle from** R-loxP and m*Klf9*TG **mice (n = 6/group). (m) Western blotting analysis of GAS muscle total protein extracts prepared from indicated mice treated for 24 h with or without Colchicine (Col) (left). Quantification of LC3-II/LC3-I (right) (n = 6/group) . (n)** Quantitative PCR analysis of mRNA levels of fatty acid oxidation and mitochondrial biogenesis related genes in skeletal muscle from R-loxP and m*Klf9*TG mice at age of 3 months (n = 6/group). (**o**) Relative mtDNA levels of skeletal muscle from R-loxP and m*Klf9*TG mice at age of 3 months (n = 6/group). (**p**) Representative electron micrographs of the soleus muscle showing mitochondria in sections from 12-week-old R-loxP and m*Klf9*TG mice (n = 6 mice/group). The left scale bar represents 2 μm and the right scale bar represents 0.5 μm. **(q) Representative image (left) and quantification (right) of the NADH-TR staining of the SOL and TA muscles of R-loxP and m*Klf9*TG mice (n = 6/group). (r) Relative mRNA levels of type I, IIA, IIX or IIB muscle markers (myosin heavy chain [Myh] 7, Myh2, Myh1 and Myh4) in the SOL (top) and TA (bottom) muscles of R-loxP and m*Klf9*TG mice (n = 6/group). All data are shown as mean ± SEM. unpaired two-tailed Student’s t tests were performed in (b, d-g, l, n, o, q and r). One-way ANOVA was performed in m. Scale bars: 20 μm.**

**Figure S2. Generation and characterization of skeletal muscle-specific Klf9-knockout mice**

(**a**) Generation of skeletal muscle-specific Klf9-knockout (*Klf9*^mlc-/-^) mice. *Klf9*^fl/fl^ mice were generated by the CRISPR/Cas9 system to insert two loxP sites into exon1 of the Klf9 gene. These mice were subsequently bred with MLC-Cre transgenic mice to obtain *Klf9*^mlc-/-^ mice. (**b**) Quantitative PCR analysis of mRNA levels of Klf9 in skeletal muscle and heart from *Klf9*^fl/fl^ and *Klf9*^mlc-/-^ mice at age of 4 months (n = 6/group). (**c**) Representative Western blotting analysis of KLF9 in skeletal muscle and heart from *Klf9*^fl/fl^ and *Klf9^mlc-/-^* mice at age of 4 months. **Quantification of the KLF9/Tubulin signal ratios (upper) (n = 6/group).** (**d**) Daily food intake of *Klf9*^fl/fl^ and *Klf9*^mlc-/-^ mice (n = 6/group). (**e**) Skeletal muscle tissue weight of *Klf9*^fl/fl^ and *Klf9*^mlc-/-^ mice (right) (n = 6/group). (**f**) Tissue weight of Liver, BAT, IW and EW from *Klf9*^fl/fl^ and *Klf9*^mlc-/-^ mice (n = 6/group). (**g**) Quantification of the myofiber cross section area size of TA from *Klf9*^fl/fl^ and *Klf9*^mlc-/-^ mice using Image J software (n = 6/group). (**h**) **Quantitative PCR analysis of mRNA levels of Klf9, Mstn, MuRF1 and MAFbx in skeletal muscle from** *Klf9*^fl/fl^ and *Klf9*^mlc-/-^**mice (n = 6/group). (i) Western blotting analysis of GAS muscle total protein extracts prepared from indicated mice treated for 24 h with or without Colchicine (Col) (top). Quantification of LC3-II/LC3-I (bottom) (n = 6/group). (j)** Quantitative PCR analysis of mRNA levels of fatty acid oxidation and mitochondrial biogenesis related genes in skeletal muscle from *Klf9*^fl/fl^ and *Klf9*^mlc-/-^ mice at age of 4 months (n = 6/group). (**k**) Relative mtDNA levels of skeletal muscle from *Klf9*^fl/fl^ and *Klf9*^mlc-/-^ mice at age of 4 months (n = 6/group). (**l**) Representative electron micrographs of the soleus muscle showing mitochondria in sections from 4 month-old *Klf9*^fl/fl^ and *Klf9*^mlc-/-^ mice (n = 6/group). The left scale bar represents 2 μm and the right scale bar represents 0.5 μm. **(m) Representative image (left) and quantification (right) of the NADH-TR staining of the SOL and TA muscles of *Klf9*^fl/fl^ and *Klf9*^mlc-/-^ mice (n = 6/group). (n) Relative mRNA levels of type I, IIA, IIX or IIB muscle markers (myosin heavy chain [Myh] 7, Myh2, Myh1 and Myh4) in the SOL (left) and TA (right) muscles of *Klf9*^fl/fl^ and *Klf9*^mlc-/-^ mice (n = 6/group). All data are shown as mean ± SEM. unpaired two-tailed Student’s t tests were performed in (b, d-h, j, k, m and n). One-way ANOVA was performed in i. Scale bars: 20 μm.**

**Figure S3. Muscle Klf9 regulates systemic glucose and lipid metabolism**

1. Blood glucose levels of 6 hr-fasted *Klf9*^fl/fl^ and *Klf9*^mlc-/-^ mice at age of 4 months (n = 6/group). (**b**, **c**) Blood glucose levels during GTT (**b**) and ITT (**c**) performed in the mice in **a** (n = 6/group). (**d**) Western blotting analysis of AKT and p-AKT (Ser473), rpS6 and p-rpS6 in the skeletal muscle and 15 min after administration of insulin (1 U/kg) of indicated mice. **Quantification of the p-AKT/AKT signal ratios (upper) (n = 6/group)**. (**e**) The growth curve of *Klf9*^fl/fl^ and *Klf9*^mlc-/-^ mice fed a HFD starting at 6 weeks of age (n = 6/group). (**f**) Gross morphology of *Klf9*^fl/fl^ and *Klf9*^mlc-/-^ mice fed a HFD for 2 months. (**g**) Daily food intake of *Klf9*^fl/fl^ and *Klf9*^mlc-/-^ mice fed a HFD for 2 months **(n = 6/group)**. (**h**) Gross appearance of soleus (SOL), gastrocnemius (GAS), quadriceps (QUA) and tibialis anterior (TA) from *Klf9*^fl/fl^ and *Klf9*^mlc-/-^ mice described in **b**. (**i**) Tissue weight of soleus (SOL), gastrocnemius (GAS), quadriceps (QUA) and tibialis anterior (TA) from the indicated mice (n=6/group). (**j**) Hematoxylin and eosin staining of paraffin-embedded TA pad sections from the mice in **h**. (**k**) Gross appearance of liver, interscapular BAT, inguinal and epididymal fat pads from mice in **h**. (**l**) Tissue weight of liver, interscapular BAT, inguinal and epididymal fat pads from mice in **h** (n = 6/group). (**m**) Hematoxylin and eosin staining and Oil Red O staining of paraffin-embedded liver from the mice in **h**. (**n**) Hematoxylin and eosin staining of paraffin-embedded BAT, inguinal and epididymal fat pad sections from the mice in **h**. (**o**) Quantitative PCR analysis of Klf9, Mstn, fatty acid oxidation and mitochondrial oxidative phosphorylation genes of GAS of the mice in **h** (n = 6/group). (**p**) Hepatic triglyceride, cholesterol, serum concentrations of triglyceride and cholesterol of mice described in **h** (n = 6/group). (**q**, **r**) Blood glucose levels during GTT (**q**) and ITT (**r**) performed in the mice in **h** (n = 6/group). (**s**) Western blotting analysis of AKT and p-AKT (Ser473) in the skeletal muscle, adipose tissue and liver and 15 min after administration of insulin **(left)** (1 U/kg). **Quantification of the p-AKT/AKT signal ratios (right) (n = 6/group).** (**t**) Blood glucose levels of 6hr-fasted mice in R-loxP and mKlf9TG mice at age of 3 months (n = 6/group). (**u**, **v**) Blood glucose levels during GTT (**u**) and ITT (**v**) performed in the mice in **s** (n = 6/group). All data are shown as mean ± SEM. unpaired two-tailed Student’s t tests were performed in (**a**-**c**, **e**, **g**, **i**, **l**, **o**-**r** and **t**-**v**). **Two-way ANOVA was performed in s.** Scale bars: 50 μm.

**Figure S4. (Supplementary to Fig 4)**

**(a) Quantitative PCR analysis of mRNA levels of Klf9 and atrophy related genes from myotubes treated with Lenti-control and Lenti-*Klf9* lentivirus (n = 4/group). (b) Quantitative PCR analysis of mRNA levels of Klf9 and atrophy related genes from myotubes treated with sh-control and sh-*Klf9* lentivirus (n = 4/group). All data are shown as mean ± SEM. Unpaired two-tailed Student’s t tests were performed in (a-b).**

**Figure S5. Regulation of muscle growth in m*Klf9*TG mice via inhibition the activity of myostatin**

(**a**) Schematic representation of the basic components of dnMstn insert packaged inside recombinant AAV gene transfer vector. The vector was single-stranded, contained ITR elements from AAV serotypes 2, and was packaged in serotype 8 capsids. (**b**) At 6 weeks of age, R-loxP and m*Klf9*TG mice received a single adeno-associated virus–mediated GFP (AAV-GFP) as control or adeno-associated virus–mediated myostatin (Mstn) inhibitor (AAV-dnMstn) through i.v. injection, and the terminal endpoint was 18 weeks of age (n = 5/group). Quantitative PCR analysis of mRNA levels of dnMstn in the liver of AAV treated R-loxP and m*Klf9*TG mice at age of 18 weeks old (n = 5/group). (**c**) Representative Western blotting analysis of phosphorylation of SMAD3 in skeletal muscle from mice described in **a (top)**. **Quantification of the SMAD3/SMAD3 signal ratios (bottom) (n = 5/group)**. (**d**) Forelimb and four-limbs grip strength was tested in mice described in **a** (n = 5/group). (**e**) Skeletal muscle tissue weight of mice described in **a** (n = 5/group). (**f**) Hematoxylin and eosin staining of paraffin-embedded TA sections of mice described in **a** (left). Quantification of the myofiber cross section area size of TA of the mice in **a** using Image J software (right) (n = 5/group). **All data are shown as mean ± SEM. One-way ANOVAs were performed in b and c or two-way ANOVAs were performed in (d - f).** Scale bars: 50 μm.
