# Supplementary material for: Klf9 Loss of Function Protects Against Glucocorticoids Induced Skeletal Muscle Wasting
Source: J Cachexia Sarcopenia Muscle. 2025 Jul 28;16(4):e70020. doi: 10.1002/jcsm.70020 (PMC12301625; doi:10.1002/jcsm.70020)
Supplement: Supplementary file 2 — Table S1 List of specific primers used for real‐time quantitative PCR gene expression analysis and ChIP analysis. [file JCSM-16-e70020-s002.docx]

**Supplemental Table**

**Klf9 loss of function protects against glucocorticoids induced skeletal muscle wasting**

Yujie Zhang, Jingran Hao, Yueyao Feng, Tongtong Qiu, Jinjin Wu, Xuenan Zhou, Heng Fan, Yongsheng Chang

Correspondence: Yongsheng Chang, Tianjin 300070, China. [changys@tmu.edu.cn](mailto:changys@tmu.edu.cn)

**Table S1. List of specific primers used for real-time quantitative PCR gene expression analysis and ChIP analysis.**

| Gene symbol | Forward primer | Reverse primer |
| --- | --- | --- |
| 36B4 | 5’-GAGGAATCAGATGAGGATATGGGA-3’ | 5’- AAGCAGGCTGACTTGGTTGC-3’ |
| Klf9 | 5’-GCACAAGTGCCCCTACAGT-3’ | 5’-TGTATGCACTCTGTAATGGGCTTT-3’ |
| Mstn | 5’-CCTCCACTCCGGGAACTGAT-3’ | 5’-TGTTTCCGTGGTAGCGTGAT-3’ |
| Cpt1b | 5’-GAGTGACTGGTGGGAAGAATATG-3’ | 5’-GCTGCTTGCACATTTGTGTT-3’ |
| Acox1 | 5’-TCCAGACTTCCAACATGAGG-3’ | 5’-CTGGGCGTAGGTGCCAATTA-3’ |
| Mcad | 5’-AACACTTACTATGCCTCGATTGCA-3’ | 5’-CCATAGCCTCCGAAAATCTGAA-3’ |
| Ppargc1a | 5’-TGATGTGAATGACTTGGATACAGACA-3’ | 5’-GCTCATTGTTGTACTGGTTGGATATG-3’ |
| Cox2 | 5’-CATGAGCCGTCCCCTCACTAGG-3’ | 5’-AATCCTGGTCGGTTTGATGCTACTG-3’ |
| dnMstn | 5’-AGTGGATCTAAATGAGGGCAGT-3’ | 5’-GTTTCCAGGCGCAGCTTAC-3’ |
| Sdhb | 5’-AATTTGCCATTTACCGATGGGA-3’ | 5’-AGCATCCAACACCATAGGTCC-3’ |
| Nrf1 | 5’-GCACCTTTGGAGAATGTGGT-3’ | 5’-CTGAGCCTGGGTCATTTTGT-3’ |
| Uqcrc2 | 5’-AAAGTTGCCCCGAAGGTTAAA-3’ | 5’-GAGCATAGTTTTCCAGAGAAGCA-3’ |
| Tfam | 5’-GAAGGGAATGGGAAAGGTAGA -3’ | 5’-AACAGGACATGGAAAGCAGAT -3’ |
| MAFbx | 5’-GACTGGACTTCTCGACTGCC-3’ | 5’-TCAGGGATGTGAGCTGTGAC-3’ |
| MuRF1 | 5’-TGTGCCAACGACATCTTCCA-3’ | 5’-AACGGAAACGACCTCCAGAC-3’ |
| Ppard | 5’-GTATGCGCATGGGACTCAC-3’ | 5’-GTCTGAGCGCAGATGGACT-3’ |
| **Myh1** | **5’-GGCAGCAGCAGCTGCGGAAGCAGAGTCTGG-3’** | **5’-GAGTGCTCCTCAGATTGGTCATTAGC-3’** |
| **Myh2** | **5’-GGCACAAACTGCTGAAGCAGAGGC-3’** | **5’-GGTGCTCCTGAGGTTGGTCATCAGC-3’** |
| **Myh4** | **5’-GAGCTACTGGATGCCAGTGAGCGC-3’** | **5’-CTGGACGATGTCTTCCATCTCTCC-3’** |
| **Myh7** | **5’-GCCAACTATGCTGGAGCTGATGCCC-3’** | **5’-GGTGCGTGGAGCGCAAGTTTGTCATAAG-3’** |
| COXII (mtDNA) | 5’-CCGACTAAATCAAGCAACAGTAACA-3’ | 5’-AAATTTCAGAGCATTGGCCATAG-3’ |
| COXIV(mtDNA) | 5’-CTATGTGTATGGCCCCATCC-3’ | 5’-AGCGGGCTCTCACTTCTTC-3’ |
| Mstn chip primer | 5’-GGGATTTATTTCATTTATGA-3’ | 5’-TGTCGTCAGGATCTATGATT-3’ |
| Mstn promoter  -2000Luc primer | 5’-GGGGTACCAATATGGTTGTAAATTATAACGCAAAAGTTGTTGCTGCTTTAAACCTTTTT-3’ | 5’-CCGCTCGAGTTGTTCTTATTTCTTCTTTTTGCTTTTGAGTAATGCCAAGTGAAATATTA-3’ |
| Mstn promoter  -1000Luc primer | 5’-CGACGCGTCAGGTGTCTGCCCTCTGGTCAAAATGAGACGCTGG-3’ | 5’-CCGCTCGAGTTGTTCTTATTTCTTCTTTTTGCTTTTGAGTAATGCCAAGTGAAATATTA-3’ |
| Mstn promoter  -500Luc primer | 5’-CGACGCGTTTTTTAGCAGGGTCACAAACTCAGCTTTCTTTAAATTAAGTCAGCTATTCC-3’ | 5’-CCGCTCGAGTTGTTCTTATTTCTTCTTTTTGCTTTTGAGTAATGCCAAGTGAAATATTA-3’ |
| Mstn promoter  -410Luc primer | 5’-CGACGCGTTTTTTTCCCTCAAATATTTGTTTTAGTAACAAAA-3’ | 5’-CCGCTCGAGTTGTTCTTATTTCTTCTTTTTGCTTTTGAGTAATGCCAAGTGAAATATTA-3’ |
| Mstn promoter  -314Luc primer | 5’-GGGGTACCTTACTAACTTAAATGATAGCAAGAGTTTTAC-3’ | 5’-CCGCTCGAGTTGTTCTTATTTCTTCTTTTTGCTTTTGAGTAATGCCAAGTGAAATATTA-3’ |
| Mstn promoter  -200Luc primer | 5’-GGGGTACCGGGATTTATTTCATTTATGAAGTAGTCAAA-3’ | 5’-CCGCTCGAGTTGTTCTTATTTCTTCTTTTTGCTTTTGAGTAATGCCAAGTGAAATATTA-3’ |
| Mstn promoter  -50Luc primer | 5’-GGGGTACCCTTGTCTCCTCTAAGTTGGAATATAAAAAGC-3’ | 5’-CCGCTCGAGTTGTTCTTATTTCTTCTTTTTGCTTTTGAGTAATGCCAAGTGAAATATTA-3’ |
| MAFbx promoter  -3300Luc primer | 5’-CGGTACCGAGCTCTTACGCGTGTGAGGGGTTACTTACTTCCAGGA-3’ | 5’ACTTAGATCGCAGATCTCGAGCCTCTGTAGCCAGTGGCAGTG-3’ |
| MAFbx chip primer | 5’-TCCGAGACAGCTGCATCACC-3’ | 5’-TGTGCAGTGTGCTCCAGGTT-3’ |
