# Supplementary material for: Klf9 Loss of Function Protects Against Glucocorticoids Induced Skeletal Muscle Wasting
Source: J Cachexia Sarcopenia Muscle. 2025 Jul 28;16(4):e70020. doi: 10.1002/jcsm.70020 (PMC12301625; doi:10.1002/jcsm.70020)
Supplement: Supplementary file 3 — Figure S1 Generation and characterization of skeletal muscle‐specific Klf9 transgenic mice. (a) Generation of skeletal muscle‐specific Klf9‐overexpression mice. Rosa26‐LSL‐Klf9 mice were generated using the CRISPR/Cas9 system to insert the CAG‐LoxP‐STOP‐LoxP‐Klf9 cassette into the mouse Rosa26 locus. These mice were subsequently bred to MLC‐Cre transgenic mice to obtain mKlf9TG mice, leading to skeletal muscle‐specific Klf9 overexpression within the skeletal muscle. (b) Quantitative PCR analysis of mRNA levels of Klf9 in skeletal muscle and heart from R‐loxP and mKlf9TG mice at age of 3 months (n = 6/group). (c) Representative Western blotting analysis of KLF9 in skeletal muscle and heart from 3‐month old R‐loxP and mKlf9TG mice. Quantification of the KLF9/Tubulin signal ratios (upper) (n = 6/group). (d) Daily food intake of R‐loxP and mKlf9TG mice (n = 6/group). (e) Skeletal muscle tissue weight of R‐loxP and Klf9TG mice (n = 6/group). (f) Tissue weight of Liver, BAT, IW and EW from R‐loxP and mKlf9TG mice (n = 6/group). (g) Quantification of the myofiber cross section area size of TA from R‐loxP and mKlf9TG mice using Image J software (n = 6/group). (h) Volcano plot of differentially expressed genes in skeletal muscles of R‐loxP and mKlf9TG mice (Down: p < 0.01 and log2FC < −2; Up: p < 0.01 and log2FC > 2). KEGG analysis of up‐regulated (i) and down‐regulated (j) differentially expressed genes in skeletal muscle from R‐loxP and mKlf9TG mice. (k) Heatmap of differentially expressed genes in skeletal muscle of R‐loxP and mKlf9TG mice. (l) Quantitative PCR analysis of mRNA levels of Klf9, Mstn, MuRF1 and MAFbx in skeletal muscle from R‐loxP and mKlf9TG mice (n = 6/group). (m) Western blotting analysis of GAS muscle total protein extracts prepared from indicated mice treated for 24 h with or without Colchicine (Col) (left). Quantification of LC3‐II/LC3‐I (right) (n = 6/group). (n) Quantitative PCR analysis of mRNA levels of fatty acid oxidation and mitochondrial bioge [file JCSM-16-e70020-s003.pdf]

Supplementary Figures

Klf9 loss of function protects against glucocorticoids induced skeletal muscle wasting

Yujie Zhang, Jingran Hao, Yueyao Feng, Tongtong Qiu, Jinjin Wu, Xuenan Zhou, Heng Fan, Yongsheng Chang

Correspondence: Yongsheng Chang, Tianjin 300070, China. changys@tmu.edu.cn

Figure S1

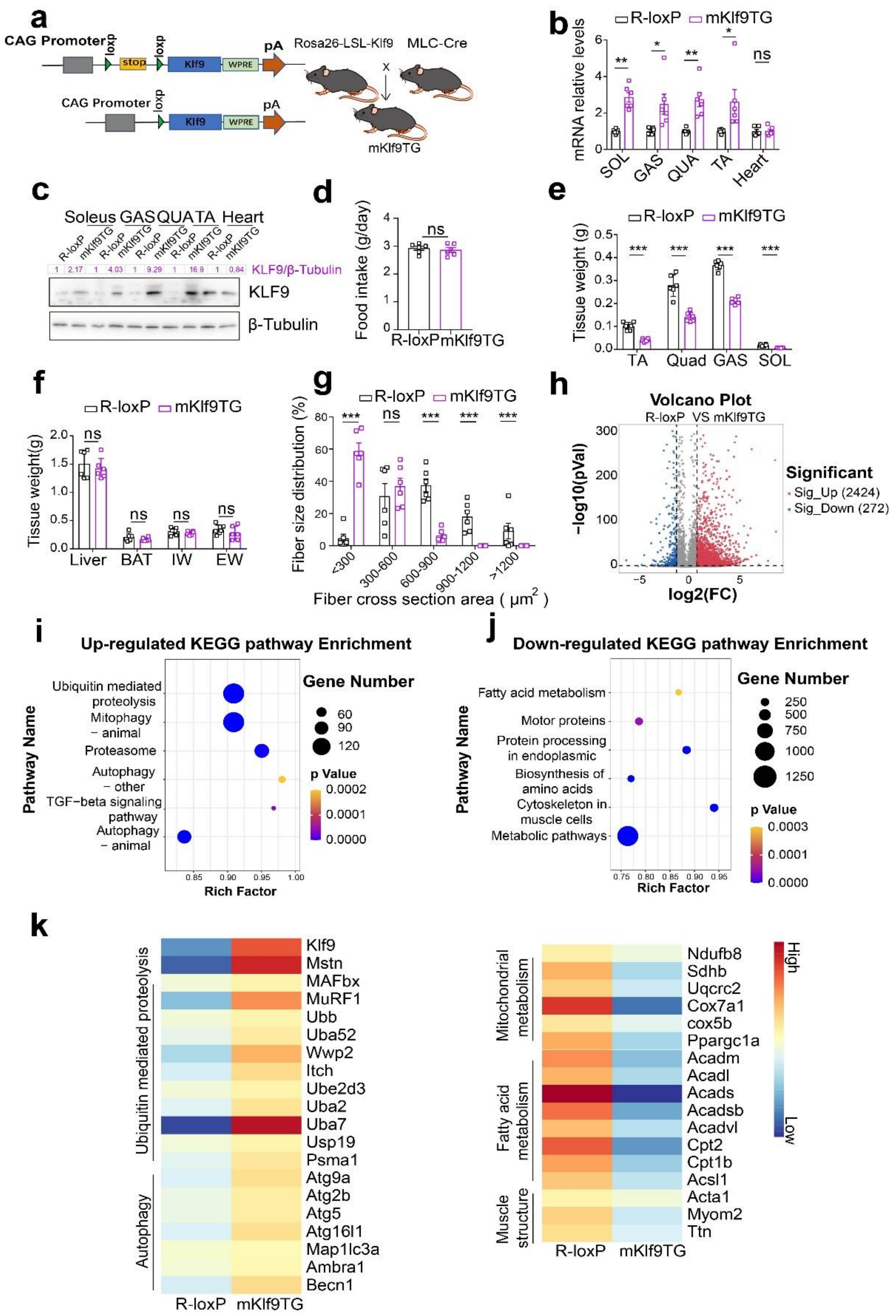

a
CAG Promoter
loxp
stop
Klf9
WPRE
pA
Rosa26-LSL-Klf9
MLC-Cre
mKlf9TG
b
R-loxP
mKlf9TG
mRNA relative levels
SOL
GAS
QUA
TA
Heart
c
Soleus GAS QUA TA Heart
KLF9/β-Tubulin
KLF9
β-Tubulin
d
Food intake (g/day)
R-loxP
mKlf9TG
e
Tissue weight (g)
TA
Quad
GAS
SOL
f
Tissue weight(g)
Liver
BAT
IW
EW
g
Fiber size distribution (%)
Fiber cross section area (μm2)
h
Volcano Plot
R-loxP VS mKlf9TG
-log10(pVal)
log2(FC)
Significant
Sig_Up (2424)
Sig_Down (272)
i
Up-regulated KEGG pathway Enrichment
Pathway Name
Ubiquitin mediated proteolysis
Mitophagy - animal
Proteasome
Autophagy - other
TGF-beta signaling pathway
Autophagy - animal
Rich Factor
Gene Number
p Value
j
Down-regulated KEGG pathway Enrichment
Fatty acid metabolism
Motor proteins
Protein processing in endoplasmic
Biosynthesis of amino acids
Cytoskeleton in muscle cells
Metabolic pathways
k
Ubiquitin mediated proteolysis
Autophagy
Klf9
Mstn
MAFbx
MuRF1
Ubb
Uba52
Wwp2
Itch
Ube2d3
Uba2
Uba7
Usp19
Psma1
Atg9a
Atg2b
Atg5
Atg16l1
Map1lc3a
Ambra1
Becn1
Mitochondrial metabolism
Fatty acid metabolism
Muscle structure
Ndufb8
Sdhb
Uqcrc2
Cox7a1
cox5b
Ppargc1a
Acadm
Acadl
Acads
Acadsb
Acadvl
Cpt2
Cpt1b
Acsl1
Acta1
Myom2
Ttn
High
Low

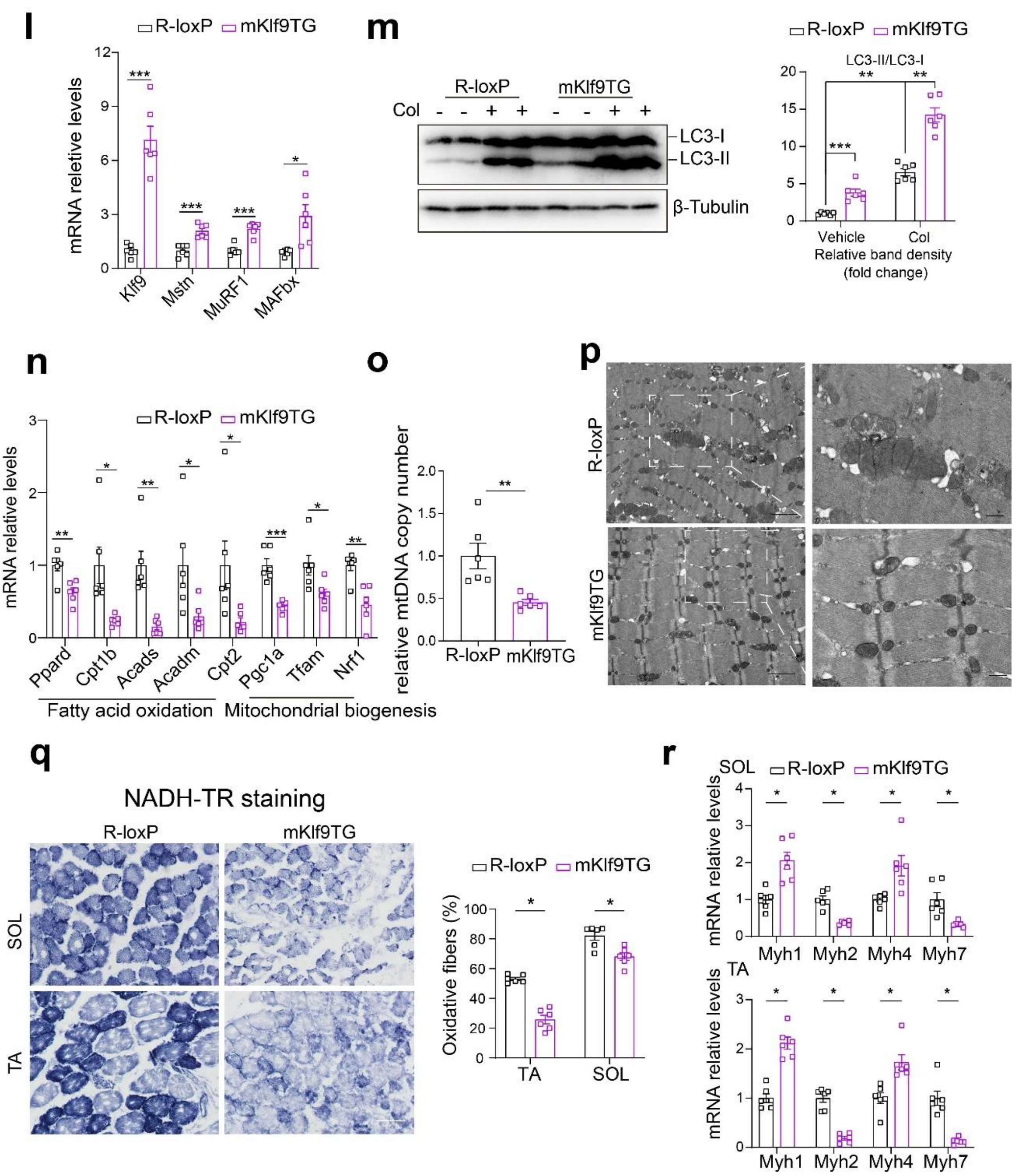

l
R-loxP
mKlf9TG
mRNA reletive levels
Klf9
Mstn
MuRF1
MAFbx
m
R-loxP
mKlf9TG
Col
LC3-I
LC3-II
β-Tubulin
LC3-II/LC3-I
Vehicle
Col
Relative band density
(fold change)
n
mRNA relative levels
Ppard
Cpt1b
Acads
Acadm
Cpt2
Pgc1a
Tfam
Nrf1
Fatty acid oxidation
Mitochondrial biogenesis
o
relative mtDNA copy number
p
q
NADH-TR staining
SOL
TA
Oxidative fibers (%)
r
Myh1
Myh2
Myh4
Myh7

Figure S2

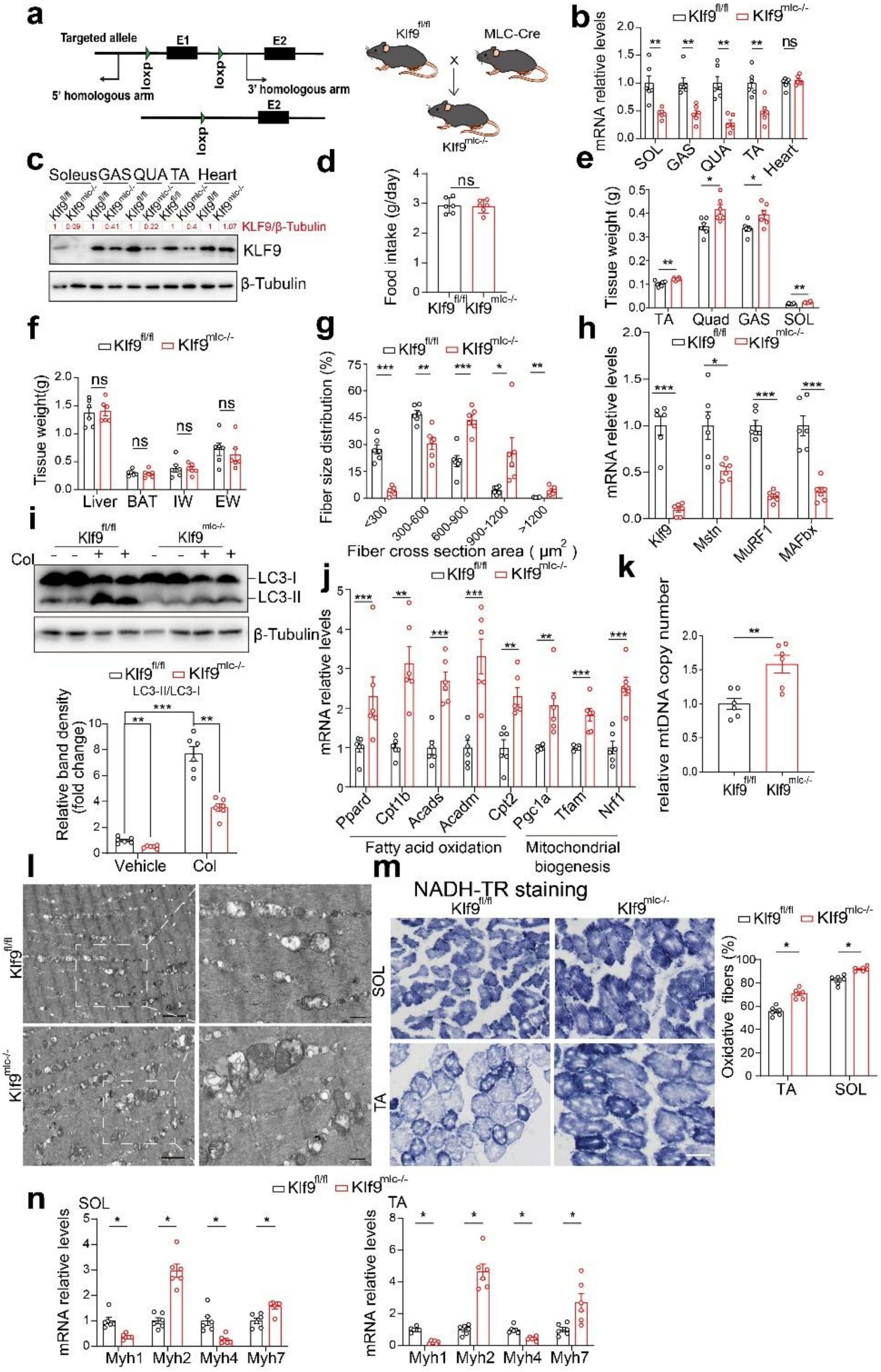
a
Targeted allele
E1
E2
loxp
5' homologous arm
3' homologous arm
Klf9 fl/fl
MLC-Cre
Klf9 mlc-/-
b
Klf9 fl/fl
Klf9 mlc-/-
mRNA relative levels
SOL
GAS
QUA
TA
Heart
ns
c
Soleus
GAS
QUA
TA
Heart
KLF9/β-Tubulin
KLF9
β-Tubulin
d
Food intake (g/day)
e
Tissue weight (g)
TA
Quad
GAS
SOL
f
Tissue weight(g)
Liver
BAT
IW
EW
g
Fiber size distribution (%)
Fiber cross section area (μm2)
<300
300-600
600-900
900-1200
>1200
h
mRNA relative levels
Klf9
Mstn
MuRF1
MAFbx
i
Col
LC3-I
LC3-II
β-Tubulin
LC3-II/LC3-I
Relative band density (fold change)
Vehicle
Col
j
mRNA relative levels
Ppard
Cpt1b
Acads
Acadm
Cpt2
Pgc1a
Tfam
Nrf1
Fatty acid oxidation
Mitochondrial biogenesis
k
relative mtDNA copy number
l
m
NADH-TR staining
SOL
TA
Oxidative fibers (%)
n
SOL
TA
mRNA relative levels
Myh1
Myh2
Myh4
Myh7

Figure S3

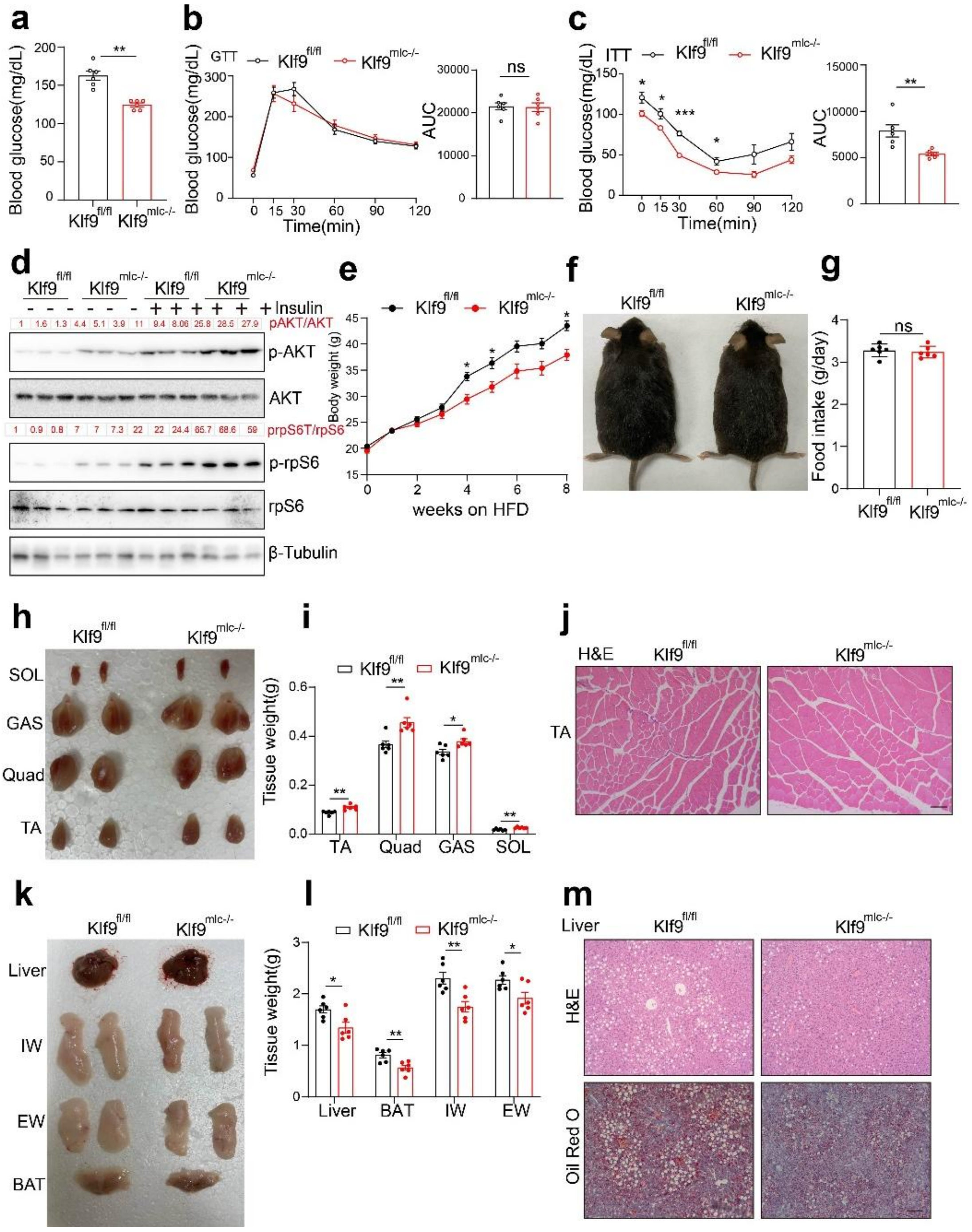
a
Blood glucose(mg/dL)
Klf9fl/fl Klf9mlc-/-
b
GTT Klf9fl/fl Klf9mlc-/-
Time(min)
AUC
ns
c
ITT Klf9fl/fl Klf9mlc-/-
Time(min)
AUC
d
Klf9fl/fl Klf9mlc-/- Klf9fl/fl Klf9mlc-/-
Insulin
pAKT/AKT
p-AKT
AKT
prpS6T/rpS6
p-rpS6
rpS6
β-Tubulin
e
Klf9fl/fl Klf9mlc-/-
Body weight (g)
weeks on HFD
f
Klf9fl/fl Klf9mlc-/-
g
Food intake (g/day)
ns
Klf9fl/fl Klf9mlc-/-
h
Klf9fl/fl Klf9mlc-/-
SOL
GAS
Quad
TA
i
Klf9fl/fl Klf9mlc-/-
Tissue weight(g)
TA Quad GAS SOL
j
H&E Klf9fl/fl Klf9mlc-/-
TA
k
Klf9fl/fl Klf9mlc-/-
Liver
IW
EW
BAT
l
Klf9fl/fl Klf9mlc-/-
Tissue weight(g)
Liver BAT IW EW
m
Liver Klf9fl/fl Klf9mlc-/-
H&E
Oil Red O

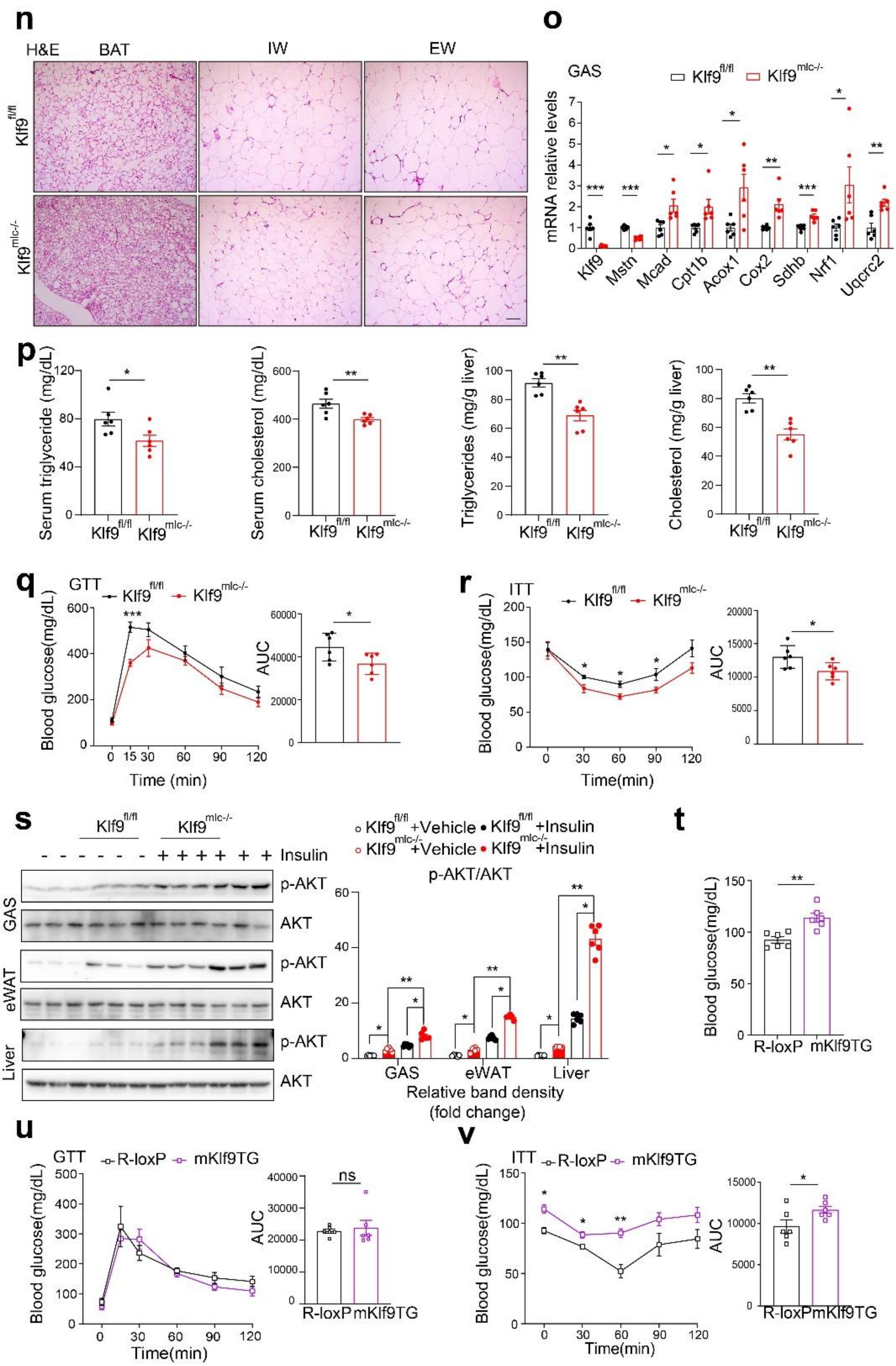
n
H&E
BAT
IW
EW
Klf9fl/fl
Klf9mlc-/-
o
GAS
Klf9fl/fl
Klf9mlc-/-
mRNA relative levels
Klf9
Mstn
Mcad
Cpt1b
Acox1
Cox2
Sdhb
Nrf1
Uqcrc2
p
Serum triglyceride (mg/dL)
Serum cholesterol (mg/dL)
Triglycerides (mg/g liver)
Cholesterol (mg/g liver)
q
GTT
Blood glucose(mg/dL)
Time (min)
AUC
r
ITT
Time(min)
s
Insulin
p-AKT
AKT
GAS
eWAT
Liver
Klf9fl/fl+Vehicle
Klf9fl/fl+Insulin
Klf9mlc-/-+Vehicle
Klf9mlc-/-+Insulin
p-AKT/AKT
Relative band density
(fold change)
t
R-loxP
mKlf9TG
u
ns
v

Figure S4

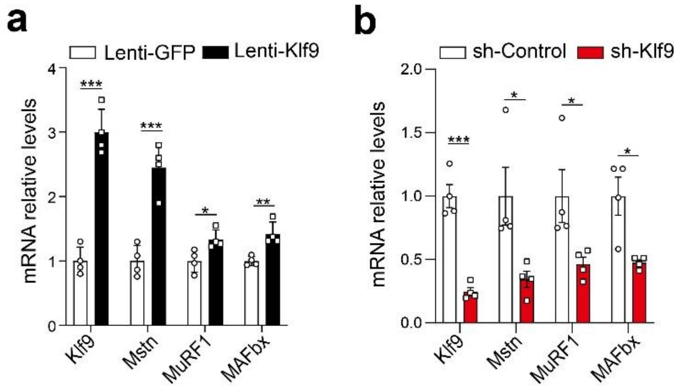
a
Lenti-GFP
Lenti-Klf9
mRNA relative levels
***
***
*
**
Klf9
Mstn
MuRF1
MAFbx
b
sh-Control
sh-Klf9
mRNA relative levels
***
*
*
*
Klf9
Mstn
MuRF1
MAFbx

Figure S5

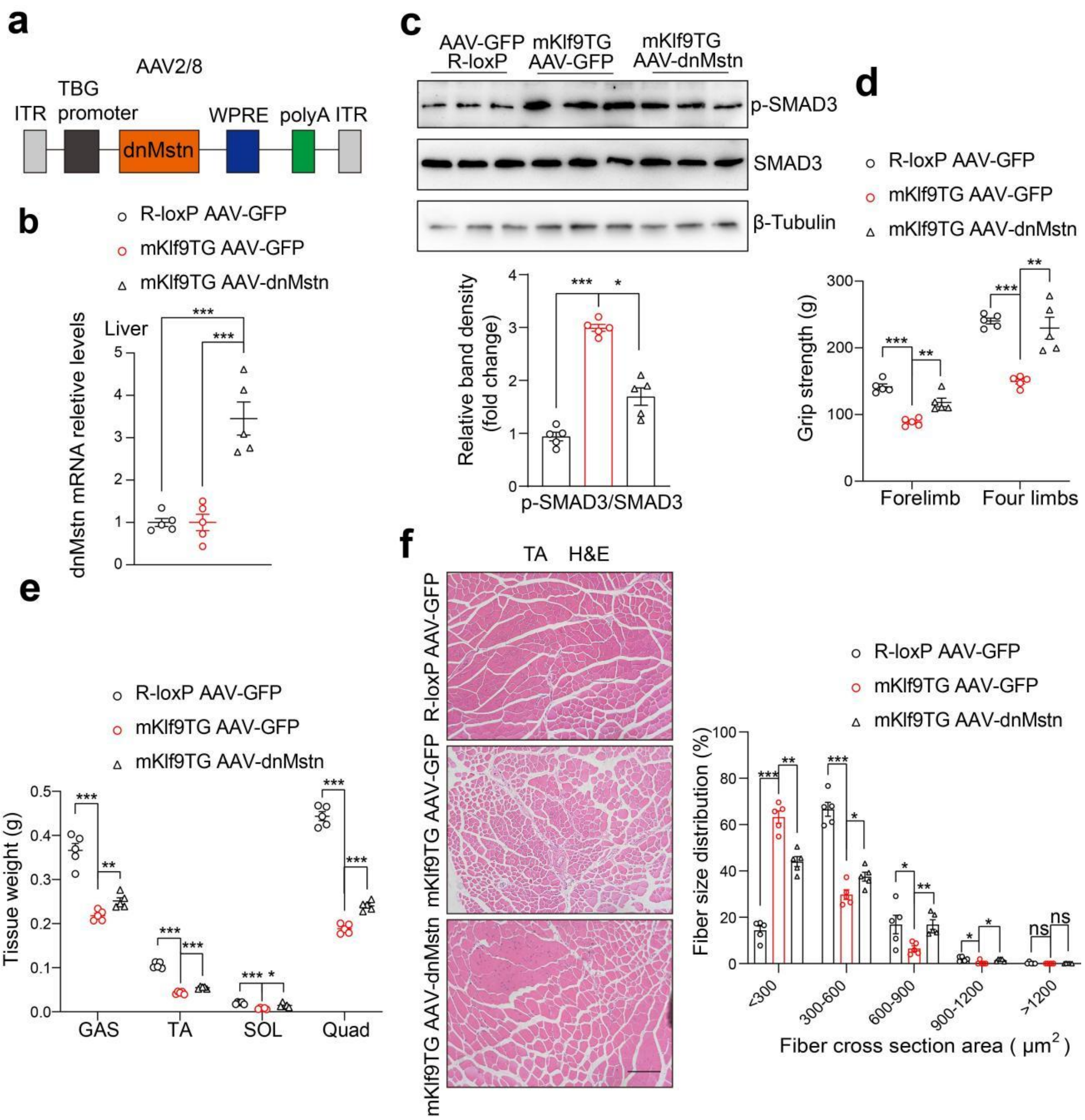

a
AAV2/8
ITR
TBG promoter
dnMstn
WPRE
polyA
ITR
b
R-loxP AAV-GFP
mKlf9TG AAV-GFP
mKlf9TG AAV-dnMstn
Liver
dnMstn mRNA reletive levels
c
AAV-GFP R-loxP
mKlf9TG AAV-GFP
mKlf9TG AAV-dnMstn
p-SMAD3
SMAD3
β-Tubulin
Relative band density (fold change)
p-SMAD3/SMAD3
d
R-loxP AAV-GFP
mKlf9TG AAV-GFP
mKlf9TG AAV-dnMstn
Grip strength (g)
Forelimb
Four limbs
e
R-loxP AAV-GFP
mKlf9TG AAV-GFP
mKlf9TG AAV-dnMstn
Tissue weight (g)
GAS
TA
SOL
Quad
f
TA H&E
R-loxP AAV-GFP
mKlf9TG AAV-GFP
mKlf9TG AAV-dnMstn
R-loxP AAV-GFP
mKlf9TG AAV-GFP
mKlf9TG AAV-dnMstn
Fiber size distribution (%)
<300
300-600
600-900
900-1200
>1200
Fiber cross section area (μm²)
